# Experimental Exploration of Hybrid Nanofluids as Energy-Efficient Fluids in Solar and Thermal Energy Storage Applications

**DOI:** 10.3390/nano13020278

**Published:** 2023-01-09

**Authors:** Humaira Yasmin, Solomon O. Giwa, Saima Noor, Mohsen Sharifpur

**Affiliations:** 1 Department of Basic Sciences, Preparatory Year Deanship, King Faisal University, Al-Ahsa 31982, Saudi Arabia; 2 Department of Mechanical Engineering, Olabisi Onabanjo University, Ago-Iwoye P.M.B. 2002, Nigeria; 3 Department of Mechanical and Aeronautical Engineering, University of Pretoria, Pretoria 0002, South Africa; 4 Department of Medical Research, China Medical University Hospital, China Medical University, Taichung 404, Taiwan

**Keywords:** coolants, efficiency, energy storage, hybrid nanofluids, phase change material, solar energy

## Abstract

In response to the issues of environment, climate, and human health coupled with the growing demand for energy due to increasing population and technological advancement, the concept of sustainable and renewable energy is presently receiving unprecedented attention. To achieve these feats, energy savings and efficiency are crucial in terms of the development of energy-efficient devices and thermal fluids. Limitations associated with the use of conventional thermal fluids led to the discovery of energy-efficient fluids called “nanofluids, which are established to be better than conventional thermal fluids. The current research progress on nanofluids has led to the development of the advanced nanofluids coined “hybrid nanofluids” (HNFs) found to possess superior thermal-optical properties than conventional thermal fluids and nanofluids. This paper experimentally explored the published works on the application of HNFs as thermal transport media in solar energy collectors and thermal energy storage. The performance of hybrid nano-coolants and nano-thermal energy storage materials has been critically reviewed based on the stability, types of hybrid nanoparticles (HNPs) and mixing ratios, types of base fluids, nano-size of HNPs, thermal and optical properties, flow, photothermal property, functionalization of HNPs, magnetic field intensity, and orientation, and *φ*, subject to solar and thermal energy storage applications. Various HNFs engaged in different applications were observed to save energy and increase efficiency. The HNF-based media performed better than the mono nanofluid counterparts with complementary performance when the mixing ratios were optimized. In line with these applications, further experimental studies coupled with the influence of magnetic and electric fields on their performances were research gaps to be filled in the future. Green HNPs and base fluids are future biomaterials for HNF formulation to provide sustainable, low-cost, and efficient thermal transport and energy storage media.

## 1. Introduction

The ever-growing global demand for energy can be linked to population explosion and economic and technological growth, which are the primary causative factors. As the major primary sources of energy, fossil fuels (coal, natural gas, and oil) consumption in various energy sectors (residential, industrial, transportation, etc.) of the global economy has led to the release of obnoxious gases and particulates into the atmosphere [1,2,3]. The aftermaths of these global actions are at present of serious concern in terms of environmental, climate, and health implications coupled with the attendant climate change and global warming. Collective efforts by most countries of the world toward ameliorating the consequences of global warming and climate change as championed by the United Nations (UN) birthed the Millennium Development Goals (2000–2015) and Sustainable Developments Goals (2015–2030). The Sustainable Development Goals (SDGs) have significantly oiled the global slogan “sustainability”, which is presently applicable to virtually all sectors of human endeavours. Sustainable manufacturing and sustainable energy are key components of the 17-point SDGs as reflected in SDG 12 and SDG 7, respectively, and are strongly connected to the nanotechnology revolution [4].

Prior to the advent of nanotechnology which spurred pioneering research in the field of heat transfer leading to the formulation of nanomaterials, such as nanofluids and nano-lubricants, cooling and lubrication of energy systems have been conducted using traditional thermal fluids (water, oil, ethylene glycol, thermal oil, transformer oil, air, etc.). For decades, studies have been performed using conventional thermal fluids in addition to surface area/volume ratio reduction, surface modification, miniaturization, and surface extension (fins) to improve heat transfer in thermal equipment. However, the thermal fluids and techniques have reached their practicable thresholds with little or no thermal improvement [5,6]. The state-of-the-art technologies in various sectors of the economy (power plants, electronics, communication, agriculture, industry, automobile, aviation, medicine, computing, space exploration, and power systems) are notable for the generation of high heat flux and have necessitated the speedy removal of such heat flux to avoid material failure culminating in system failure. These challenges call for a solution and energy-efficient fluids such as nanofluids appear to fit into this [7,8].

The low κ and coefficient of heat transfer of conventional thermal fluids led to an extensive search for energy-efficient fluids as pioneered by Maxwell [9], Ahuja [10], and Masuda et al. [11]. Behind these pioneering efforts was the intention to improve the κ and consequently the coefficient of heat transfer by adding micro-size and nano-size particles of metals and non-metals with higher κ into conventional thermal fluids. Finally, the deposition of the nano-size particles (SiO_2_, TiO_2_, and Al_2_O_3_) into water yielded higher κ compared with water [11]. The resulting energy-efficient fluids were coined “nanofluids” two and a half decades ago [12]. After 12 years of intensive studies on different types of nanofluids (NFs) in terms of various types of nanoparticles (NPs), diverse base fluids, and different thermal properties, as advanced thermal fluids in various applications, the innovative concept of combining two or more NPs at different mixing ratios (volume or mass) was introduced by Chopkar et al. [13] and Jana et al. [14]. The intention was to synergize the thermal properties of different NPs to formulate hybrid nanofluids (HNFs) with improved properties compared with mono nanofluids (MNFs).

**Figure 1:**
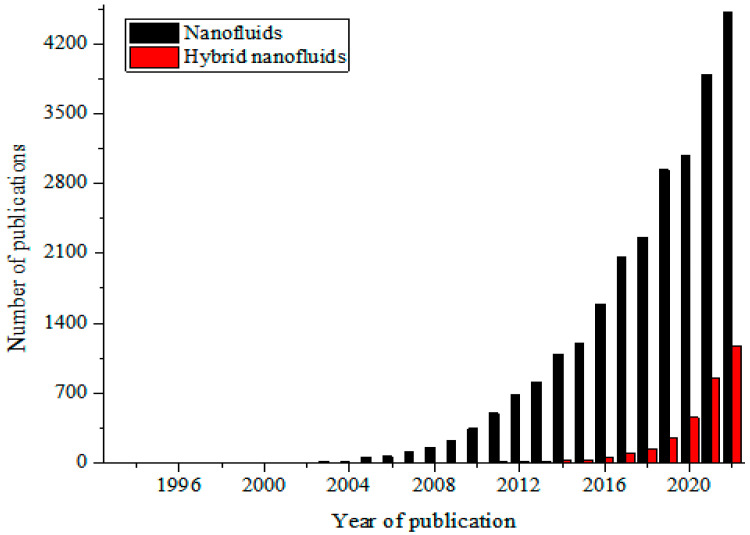
Temporal publication trend of articles on nanofluid and hybrid nanofluid studies (Source: SCOPUS (26 December 2022)).

**Figure 2:**
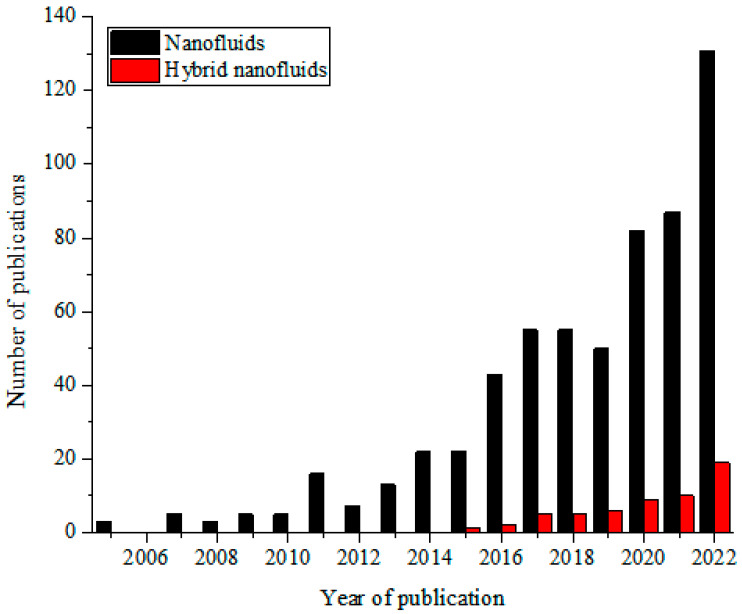
Temporal publication trend of review papers on nanofluid and hybrid nanofluid studies (Source: SCOPUS (26 December 2022)).

These MNFs are widely studied as nano-coolants (in thermal transporting equipment) [15,16,17,18,19,20,21,22,23,24,25,26], nano-lubricants (in moving and sliding parts of automobiles and machinery) [27,28,29], nano-based PCM (as energy storage materials) [30,31,32], nano-drilling mud [33,34,35], nano-CO_2_ absorbents [36,37,38], nano-water desalination and purification materials [39,40,41], nano-cutting fluids (in machining applications) [42,43,44], nano-oil recovery materials [45,46,47], nano-sensors [48,49,50], etc. The passive augmentation of the thermal properties of NFs via HNFs is presently receiving unprecedented attention with an increasing number of publications in this regard [13,14,51,52,53,54] but not as that of MNFs. Owing to the background that the HNFs offered higher thermal and convective properties compared to NFs, studies have been conducted on the various applications of HNFs similar to those of MNFs with increasingly growing publications in the open literature. In comparison to MNFs, studies are very scarce in some areas of applications of HNFs while some are witnessing an increasing number of publications in the public domain due to growing research interest. Generally, documentation on the applications of HNFs is still on the increase in the open literature. This observation is illustrated in Figure 1 (article publication trend on MNF and HNF studies) and Figure 2 (review paper publication trend on MNF and HNF studies).

The deployment of HNFs as energy-efficient fluids in solar energy and thermal energy storage to improve energy efficiency, absorption of solar energy, and PTEC performance, and reduce exergetic efficiency, have been investigated by various authors. These studies, especially the solar energy application of HNFs as energy-efficient working fluids, have been carried out using theoretical, experimental, and numerical methods, with the majority conducted using the numerical technique. However, the experimental studies are of interest to this present work. An experimental exploration of the studies available in the open literature concerning the utilization of HNFs as nano-coolants and nano-phase change materials (PCMs) in solar energy and thermal energy storage, respectively, has been presented and discussed. The thermal and optical properties of different HNFs have been compiled and presented in this work.

**Figure 3:**
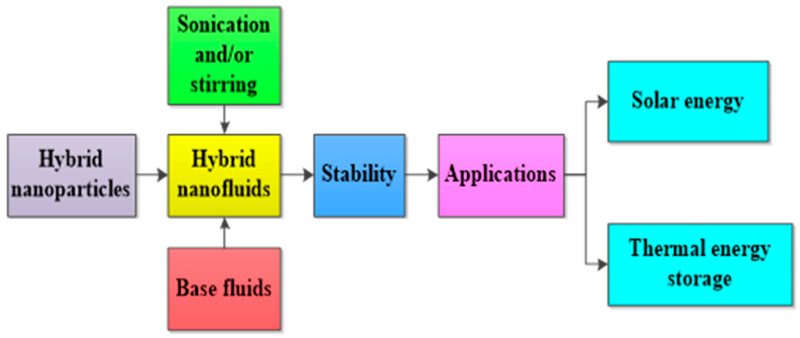
A schematic presentation of this present work.

**Figure 4:**
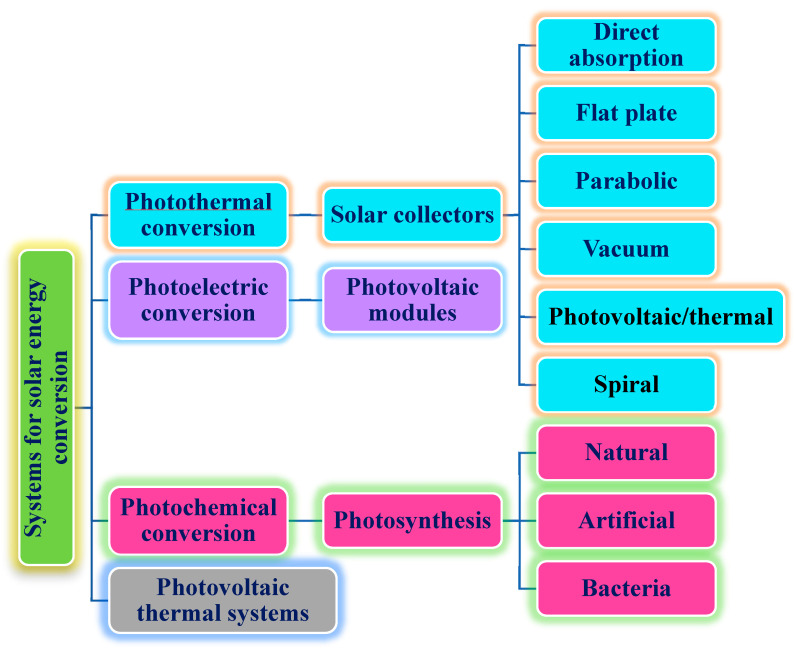
Description of solar energy conversion and systems.

In addition, the PTEC and thermal energy storage characteristics of HNFs in relevant applications have been reviewed. Novel to this review is the special focus on the effect of mixing ratios of HNPs, nano-size of HNPs, types of base fluids and HNFs, magnetic field, flow conditions, and thermal and optical properties on various parameters relevant to the HNF applications. Amongst the key parameters are the energy and exergy efficiency of solar collectors for solar applications and latent heat of fusion, melting and freezing process temperature, and thermal stability for thermal energy storage applications. A schematic representation of this review work is given in Figure 3. The solar energy conversion and systems are presented in Figure 4. An increase in review papers on HNF studies has been observed (see Figure 2) but the majority of these papers are focused on HNF preparation, thermophysical properties, and stability. A few of these papers centred on hydrodynamic fluid flow and heat transfer in selected thermal systems. However, very scarce review papers have been published on solar applications of HNFs. The few existing ones fail to provide a distinct and in-depth understanding of the study approach (numerical and experimental), discussion on energy storage (which is complementary to solar applications), and effect of magnetic field on the performance of HNFs in solar applications. These research gaps in addition to the need to provide an update on the solar application of HNFs have prompted this work.

This paper is structured into six sections. Section 1 is the general introduction of the subject, focus, and objectives of this work while Section 2 addresses the PTEC efficiency of different working fluids engaged in solar energy applications. Section 3 deals with the exergetic and energetic performance of various HNFs in different types of solar collectors. The thermal energy storage performances of different types of hybrid-based PCMs and the effect of surface modification are discussed in Section 4. The challenge, future research, and conclusion are presented in Section 5 and Section 6, respectively.

## 2. Photothermal Performance of Hybrid Nanofluids

**Figure 5:**
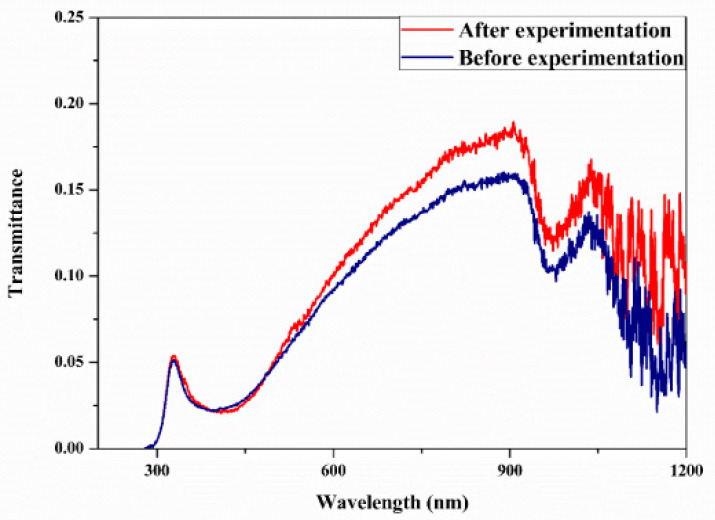
Transmittance spectral analysis of optimized 0.2 vol% ATO-AG/DIW nanofluid (before and after the experiment) for DASC application (Adapted from Sreekumar et al. [63]).

**Figure 6:**
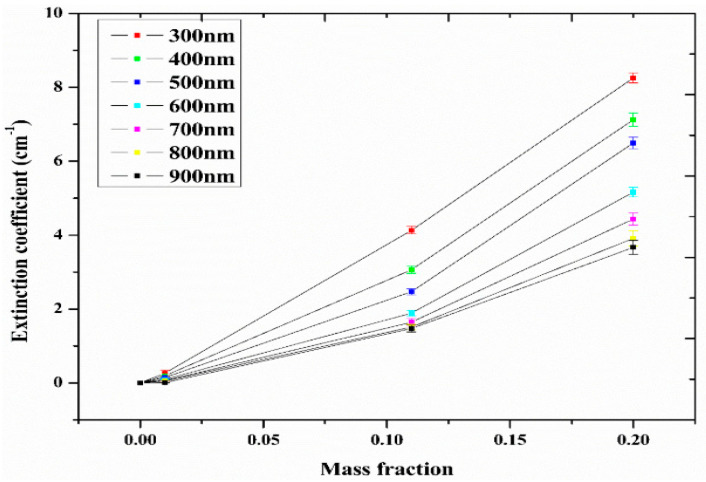
Extinction coefficient of ATO-Ag/DIW nanofluid as a function of mass fraction under varying wavelengths (Adapted from Sreekumar et al. [63]).

**Figure 7:**
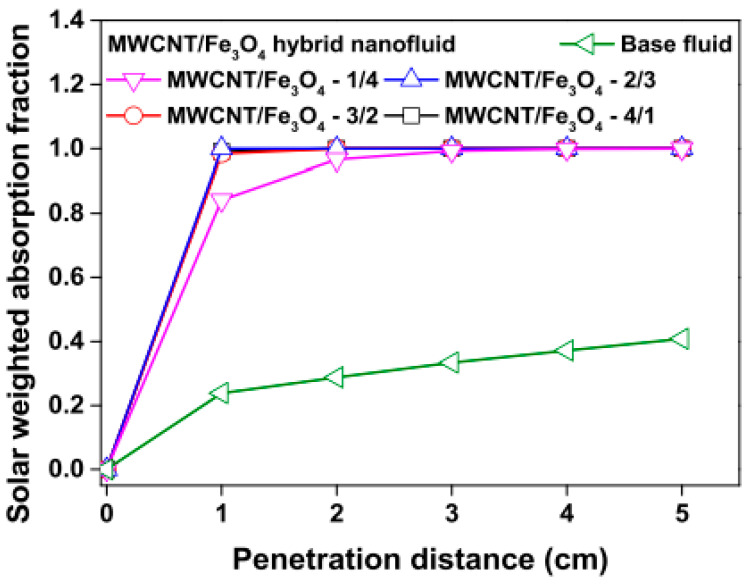
Effect of increasing penetration distance on solar weighted absorption fraction of MWCNT/Fe_3_O_4_ nanofluids and base fluid (Adapted from Tong et al. [61]).

**Figure 8:**
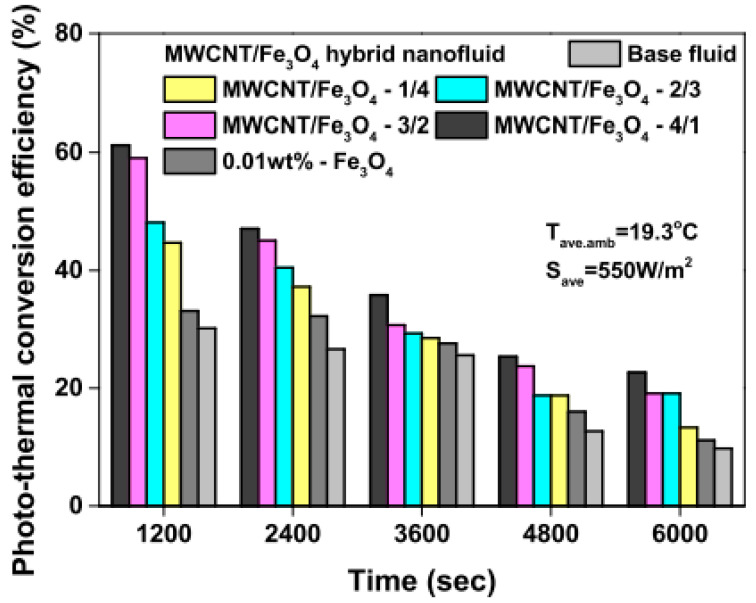
Photothermal energy conversion efficiency of MWCNT/Fe_3_O_4_ nanofluids and base fluid with increasing irradiation (Adapted from Tong et al. [61]).

Studies on the thermal-optical properties (κ, μ, c_p_, rheology, transmittance, thermal diffusivity, sun intensity, absorbance, and extinction coefficient) of HNFs (with and without the influence magnetic field) at different volume/weight concentrations, temperatures, and shear rates (where applicable) as working fluids in solar energy applications are presented in Table 1. In addition, various NPs (with different nano-sizes) and base fluids (DW, EG, PG, vegetable oil, etc.) used in the formulation of HNFs at different mixing ratios and process strategies (1- or 2-step), along with the different stability tests (ZP, DLS, UV, visual, and pH) and surfactants (PVP, SDS, SHMP, gum Arabic, etc.) used to achieve stable HNFs (where engaged), are compiled in Table 1. Additionally, short remarks on the key findings from the various studies compiled in this regard were provided in the table. These papers were notable studies found in the open domain and were selected based on the different base fluids, HNPs, temperatures, nanoparticle size, surfactants, HNF processing strategies, mixing ratios, properties (thermal-optic), stability tests, and deployment of the magnetic field. In Figure 5, Figure 6, Figure 7 and Figure 8, the spectral transmittance, EC, SWEA fraction, and PTEC of different HNFs are presented. The transmittance of 0.2 vol% ATO-AG/DIW NF as a function of wavelength for DASC is presented in Figure 5. The wavelength is observed to be directly related to the transmittance of the HNF. Figure 6 presents the EC of ATO-AG/DIW NFs as a direct function of mass fraction and an indirect function of wavelength while Figure 7 shows the influence of penetration distance on SWEA for ATO-AG/DIW NFs. The effect of irradiance time on the PEC efficiency of MWCNT-Fe_3_O_4_ NFs is shown in Figure 8. The addition of different kinds of HNPs at various mixing ratios into diverse base fluids is known to produce different HNFs with varying thermal and optical properties [55,56,57,58]. This is because the individual base fluid and NPs have different absorption capacities (at different wavelengths) and thermal properties lending to synergetic effects on these properties. A shift in the wavelength range (visible and near-infrared) and the peak is mostly observed due to the mixing of different NPs [59,60,61,62]. For broadband absorption to be achieved, a combination of diverse absorption peak HNFs was engaged.

Table 2 gives a summary of the thermo-optical properties, formulation materials, process methods, and photothermal performances of different HNFs used as working fluids in solar energy applications. Details of the various HNPs (with different nano-sizes) and base fluids used to formulate HNFs at diverse volume/weight concentrations, mixing ratios, and process strategies are presented. Additionally, the various stability tests to monitor HNF homogeneity and the surfactants deployed to ensure HNF stability are compiled in Table 1. In addition, remarks on the important findings from the reviewed papers used to compile the table are included. These papers were notable studies carried out from 2015 to 2022 and based on the different base fluids, HNPs, nanoparticle sizes, surfactants, HNF process methods, mixing ratios, thermo-optical properties, photothermal performance, stability tests, and use of the magnetic field. The effects of volume fraction (0.002–0.15%) and core size (20 nm and 25 nm) of TiO_2_-Ag/DIW NFs as working fluids in a solar collector were explored by Xuan et al. [64]. The work was performed by exposing the test samples to the sun and the PTEC efficiency was estimated by substituting all applicable and measured parameters into an empirical equation. They reported that increasing the core size and volume fraction (to 0.1%) favored the improvement of the absorbed energy and consequently enhanced the temperature and PTEC efficiency of HNFs. With an increment of energy absorptance as the volume fraction increased and the temperature also increased as the solar radiation increased. Absorbed energy, temperature, and thermal efficiency of 57.89, 390.88, and 413.36 W/m^2^; 60.21 °C, 66.65 °C, and 66.93 °C; and 16.07%, 20.86%, and 20.9% were obtained for TiO_2_, Ag, and TiO_2_-Ag NFs, respectively, against temperature (57.52 °C) and thermal efficiency (15.52%) of DIW. Increasing the volume fraction from 0.005% to 0.01% enhanced energy absorbed from 0.76 to 0.93, with maximum temperature occurring at 0.01% concentration for TiO_2_-Ag NF.

**Table 1:** Different HNPs, base fluids, surfactants, stability tests, process methods, and thermo-optical properties related to HNFs use in solar energy applications.

References	HNF (Mixing Ratio)/Base Fluid	*φ*	Temperature (°C)	Nano-Size (nm)	Stability (Tests, and Surfactants)	Properties	Result
Mechiri et al. [65]	Cu-Zn/ground nut (50:50, 75:25,25:75)	0.1–0.5 vol%	30–60	25	ZP, surfactant, (2-step)	κ and μ	Highest κ and μ for Cu-Zn/oil (50:50). Newtonian flow for oil and HNFs.
Chandran et al. [66]	ZnO-paraffin wax /PG-DIW	4–16 wt% (ZnO-paraffin) and 2 vol% wt% (HNFs)	30	ZnO–30-45	(2-step)	κ and c_p_	Maximum enhancements of κ = 10.4%, cp = 18.7%, heat transfer = 13.54%, and coefficient of heat transfer = 15.37%. HNF with 10 wt% paraffin and 2 vol% ZnO yielded κ = 3.5%, c_p_ = 5.1%, and coefficient of heat transfer = 15.37%.
Akilu et al. [67]	SiC-CuO/C/EG (8:2)	0.8–3.13 wt% (0.25–0.99 vol%)	25–80	SiC-29, CuO/C-28.5, SiC-CuO/C -12-28	ZP, DLS, Visual, PVP (2-step)	Rheology, κ, and μ.	At 3.13 wt% and 80 °C, κ and μ were enhanced by 19.3% and 205%, respectively, in comparison with EG. Newtonian behavior was observed for the HNFs at 50–250 s^−1^.
Ghafurian et al. [68]	MWCNT-GNP/seawater (50:50)	0.001–0.04 wt%	sonication time (30–240)	GNP-40, MWCNT-20-30	ZP, UV, pH, Gum Arabic (2-step)	Sun intensity, pH, absorbance.	At optimum sonication time of 120 min, the maximum solar evaporation efficiency (61.3%) and evaporation rate (2.89 kg/m^2^ h), and lowest average particle size were achieved when *φ* = 0.01 wt% and at the solar intensity of 3.6 suns.
Asadi et al. [69]	MgO-MWCNT/EO (80:20)	0.25–2 wt%	20–50	MgO-30nm MWCNT-20-30nm	(2-step)	Κ	Maximum enhancement of 65% at 50 °C and *φ* = 2 wt%.
Gugulothu and Pasam [70]	CNT-MoS_2_/sesame oil (1:2)	0.5–3 wt%	20–50	CNT-30 MoS_2_-30	SDS (15%), Visual (2-step)	κ, μ, and c_p_	Maximum κ (28.31%), c_p_ (10.98%), and μ were achieved at *φ* = 3 wt% as compared with sesame oil.
Kumar et al. [71]	ZnFe_2_O_4_/DW	0.02–0.5 wt%	30–80	-	UV, Visual, CTAB (2-step)	κ and μ.	The highest κ was 11.8% for 0.5 wt at 80 °C.
Tong et al. [61]	MWCNT-Fe_3_O_4_/ EG-W (20:80)	0.02 wt%	20–50	MW-10-20 Fe_3_O_4_-10	ZP and V	κ	κ = 0.541 W/m°C (Fe_3_O_4_ @ 0.2 wt%) and κ = 0.562 W/m°C (MWCNT-Fe_3_O_4_ (80:20) @ 0.01 wt%) at 50 °C.
Ali et al. [72]	Al_2_O_3_-TiO_2_/5W-30	0.1 vol% (0.05% Al_2_O_3_ + 0.05% TiO_2_ + 1.9 wt% oleic)	-	Al_2_O_3_-8-12 TiO_2_-10	Visual	κ and μ	κ was enhanced by 7–11% relative to the base oil. Non-Newtonian and pseudoplastic behavior were observed.
Mendari et al. [73]	Al_2_O_3_-CuO/EG-DW (50:50) and EG	0.001% CuO and 0.04% Al_2_O_3_	-	Al_2_O_3_-40 CuO-100	UV, Visual, pH, SHMP (2-step)	EC, pH, and absorbance	Absorbency and EC of the Al_2_O_3_-CuO NFs were close to the sum of the individual NPs in the HNF. EC of EG-DW-based HNFs was higher than EG-based HNFs.
Mendari et al. [74]	CuO-Al_2_O_3_/DW	0.001% CuO and 0.04% Al_2_O_3_	-	Al_2_O_3_-40 CuO-100	UV, Visual, pH, SHMP (2-step)	EC, pH, μ, and absorbance	Absorbency and EC of the Al_2_O_3_-CuO NFs were close to the sum of the individual NPs in the HNF. EC and absorbance improved with volume fraction.
Mendari et al. [75]	Al_2_O_3_-CuO/EG-DW (50:50) and DW	0.001% CuO and 0.04% Al_2_O_3_	-	Al_2_O_3_-40 CuO-100	UV, Visual, pH, SHMP (2-step)	Absorbance and κ	Stability and κ of EG-DW and DW-based Al_2_O_3_-CuO NFs were strongly related to sonication time, pH, and surfactant mass fraction. The HNFs were stable at peak absorbance and κ values.
Mendari and Alemrajabi [76]	Al_2_O_3_-CuO/EG-DW (50:50), EG, and DW	0.001% CuO and 0.04% Al_2_O_3_	-	Al_2_O_3_-40 CuO-100	UV, Visual, pH, SHMP (2-step)	EC and Absorbance	Absorbency and EC of the Al_2_O_3_-CuO NFs were close to the sum of the individual NPs in the HNF. EC of DW-based HNFs was higher than EG-DW and EG-based HNFs.
Shin et al. [77]	MWCNT-Fe_3_O_4_ (1:1)/EG-W (20:80 wt%)	0.005–0.2 wt%	-	-	Visual (2-step)	B = 250–750 G, κ,	κ enhanced as *φ* and magnetic field intensity increased. Maximum κ of 0.562, 0.580, and 0.569 W/m °C for Fe_3_O_4_, MWCNT, and MWCNT/Fe_3_O_4_ NFs (absence of magnetic field) and 0.583 and 0.59 W/m°C for Fe_3_O_4_ and MWCNT/Fe_3_O_4_ NFs (under the magnetic intensity of 750 G), respectively, at *φ* = 0.2 wt% and 50 °C.
Harikrishnan et al. [78]	CuO-TiO_2_ (50:50)/paraffin	0.25–1 wt%	-	CuO-TiO_2_- 21	SDBS, Visual	κ, μ	κ and μ of CuO, TiO_2_, and CuO-TiO_2_ NF were enhanced by 51.5%, 32.3%, and 46.81%, and 7.76%, 4.85%, and 6.15%, respectively, at 60 °C.
Ali et al. [79]	Cu-GNP/EO (5W-30)	0.03–0.6 wt%	-	Cu-10-20 GNP-5-10 μm	UV, Visual, Oleic (2 wt%) (2-step)	μ	μ of Cu-GNP/EO NFs enhanced with *φ* from 54.3–55.2 mm^2^/s and 9.4–10 mm^2^/s at 40 °C and 100 °C, respectively.
Ali et al. [80]	Al_2_O_3_-TiO_2_ (0.05:0.05 wt%)/5W-30	1.5–1.95 wt% and 0.05–0.5 wt% for OA	-	Al_2_O_3_- 8-12 TiO_2_- 10	UV	μ	μ was 54.06, 54.01 and 51.62 mm^2^/s and 9.45, 9.42, and 9.23 mm^2^/s, for EO, EO + OA, and 0.1 wt% Al_2_O_3_-TiO_2_ NF at 40 °C and 100 °C, respectively. The viscosity index of 160, 160, and 163 were obtained for EO, EO + OA, and 0.1 wt% Al_2_O_3_-TiO_2_ NF, respectively.
Parameshwaran et al. [81]	Ag-TiO_2_/organic ester	0.1–1.5 wt%	-	Ag-TiO_2_- 10-95	-	μ and κ	With increasing *φ*, κ increased from 0.286 W/m K to 0.538 W/m K translating to 10–52% enhancement. μ was enhanced by 0.35–3.8% for the HNFs. Newtonian behavior was demonstrated by the HNFs,
Parameshwaran et al. [82]	Cu-TiO_2_/pristine	0.02–0.1 wt%	-	-	PVP and ethanol (2-step)	κ	κ was augmented up to 0.08 wt% (0.1926 W/m K) translating to an enhancement of 5.53%.
Li et al. [83]	β-CD-TiO_2_-Ag/ EG-DIW (40:60)	0.025–0.1 vol%	-	β-CD-TiO_2_-Ag–40-50 TiO_2_-Ag–40-50 TiO_2_ -25-30	ZP	κ	κ enhanced as *φ* increased with an improvement of 24.58–42.17% for *φ* = 0.1 vol% at 20–50 °C.
Nithiyanantham et al. [84]	SiO_2_-Al_2_O_3_/binary nitrate salt (eutectic)	1 wt%	-	SiO_2_-Al_2_O_3_–12, 14, 17	-	μ, κ, thermal diffusivity	At temperatures of 250–400 °C, the thermal diffusivity, κ, and μ of 35-SiO_2_-Al_2_O_3_ nano-PCM were augmented by 7–14%, 11–19%, and 25–34%, respectively, compared with eutectic-based PCM.
Sundar et al. [85]	ND-Co_3_O_4_ (67:33)/DW	0.05–0.15 wt%	20–60	-	Visual	μ and κ	For 0.05–0.15 wt% and at a temperature range of 20–60 C, the κ and μ were enhanced by 2.07–15.71% and 6.96–45.83% compared with DW.

Using DIW-based HNFs (Au (0.5–2.5 ppm) + Ag (0.15 and 0.5 ppm)) as thermal fluids, Chen et al. [86] studied the absorptance, SAR, and photo-thermal performance in DASCs. This work was carried out using simulated solar intensity and a theoretical model (based on energy balance) was developed to evaluate the PTEC efficiency. They observed that the temperature change of DIW-based Au (1.75 ppm) + Ag (0.15 ppm) NF was 15.61% and 8.98% higher than those of Ag (0.15 ppm) and Au (1.75 ppm) NFs, respectively. Increasing the concentration of Au in the DIW (45 °C) was noticed to improve the temperature of Au/DIW (2.5 ppm) NF (64 °C). The PTEC efficiency of 30.97%, 19.01%, and 11.90% was obtained for Au (1.75 ppm) + Ag (0.15 ppm), Au (1.75 ppm), and Ag (0.15 ppm) NFs respectively. The PTEC efficiency of the HNF translated to the sum of the individual efficiency of the MNFs. It was observed that the estimated (43.72 W/μL) and predicted SAR (43.81 W/μL) of Au (1.75 ppm) + Ag (0.15 ppm) NF based on the individual SAR values of Au (1.75 ppm) and Ag (0.15 ppm) NFs and PTEC performance of the HNF were almost equal. Carrillo-Torres et al. [87] studied the thermal stability and PTEC efficiency of Au-Ag NPs dispersed in water as thermal fluid in solar collectors. This experiment was conducted by exposing the test samples to laser light and the PTEC efficiency was calculated using the existing energy balance model. They reported that temperature change was enhanced as irradiation time, optical density, and heating profile increased. Using the HNF, maximum PTEC efficiency of 74.68% was obtained while a temperature of 20 °C was recorded after exposing the HNF to 15 min of irradiation. Furthermore, the HNF showed no significant change in optical properties after 12 h exposure to irradiation and 10 cycles of cooling/heating, thus, indicating thermal stability.

Chen et al. [88] examined the effect of mixing ratio (1:9–9:1) and *φ* (0.02–0.12 vol%) on the SWEA and PTEC efficiency of DIW-based CuO-ATO NFs in a solar collector. Alaboratory-based simulator was used in the experiment while the PTEC efficiency was evaluated based on the energy balance during the testing process. The temperature change, absorption coefficient, transmittance, SWEA fraction, and PTEC efficiency were noticed to be strongly related to the mixing ratio and *φ* of the HNFs. Increasing the solar radiation exposure of CuO-ATO NFs was noticed to increase temperature change until 7000 s, after which a decline was observed. At an optical distance of 1 cm, optimum absorption coefficient, transmittance, and SWEA fraction were attained with 0.12 vol% CuO-ATO (4:6) NF whereas optimum temperature change and thermal efficiency were reached using 0.1 vol% CuO-ATO (4:6) NF. Peak SWEA fraction, temperature change, and PTEC efficiency of 99.6%, 43.6 °C, and 92.5% were obtained for CuO-ATO NFs against 89.5%, 39.8 °C, and 81.3% and 89.8%, 39.6 °C, and 80.7% recorded for CuO and ATO NFs, respectively. Menbari et al. [75] deployed water and EG-W-based Al_2_O_3_-CuO NFs as working fluids in a DASC and examined the influence of base fluid type (water and EG-water), *φ* (0.002–0.008% (CuO) and 0.05–0.2% (Al_2_O_3_)), and volume flow rate (10–100 L/h) on the thermal efficiency. In addition, stability parameters (surfactant mass fraction, sonication time, and pH) on the absorbance and κ of the HNFs were measured. Their results demonstrated that stable HNFs were observed at high and low values of κ and absorbance. An increase in flow rate caused a reduction in temperature difference and outlet temperature while it enhanced inlet temperature and thermal efficiency. A rise in *φ* was demonstrated to improve temperature change, solar irradiation, and thermal efficiency. Both the solar irradiation and thermal efficiency of the collector were noticed to be higher for water-based HNFs than EG-water-based HNFs. 

Khashan et al. [89] experimented the PTEC efficiency of DIW and DIW-kerosene-based Fe_3_O_4_-SiO_2_ NFs (1 mg/mL and 2 mg/mL) as thermal fluids in a solar collector. The test samples were exposed to irradiance via a solar similar and the estimation of the PTEC efficiency was carried out using the energy balance empirical equation. Results showed that after 10 min exposure of DIW, Fe_3_O_4_ (1 mg/mL), and Fe_3_O_4_-SiO_2_ (1 mg/mL) NFs to solar irradiation, the difference in temperature between the top and bottom surface of the collector was 1.4 °C, 2.7 °C, and 3.2 °C, respectively. After 65 min of irradiation, the surface temperature of DW and Fe_3_O_4_-SiO_2_ (1 mg/mL) NF increased by 9.2 °C and 12.7 °C, respectively. This was due to an increase in the absorption capacity of Fe_3_O_4_-SiO_2_ NF. At different collector heights and after 10 min of irradiation, PTEC efficiency of 10–17%, 27–83%, and 30–89% was obtained using DW, Fe_3_O_4_, and Fe_3_O_4_-SiO_2_, respectively. With 300 min of irradiation on the Fe_3_O_4_-SiO_2_ (1 mg/mL) NF + kerosene mixture, the part with kerosene alone and that with Fe_3_O_4_-SiO_2_ (1 mg/mL) NF recorded top and bottom temperatures of 50.7 °C and 47.8 °C and 57.3 °C and 55.5 °C, respectively. Additionally, after 5 min of irradiation, PTEC efficiency of 65.6%, 85.4%, and 98.5% was attained with DIW, kerosene + 2 mg/mL Fe_3_O_4_-SiO_2_ NF, and kerosene + 1 mg/mL Fe_3_O_4_-SiO_2_ (1 mg/mL) NF, respectively. The authors recommended similar future research using kerosene-based HNFs.

The utilization of DIW-based CuO-ZnO (70:30 and 50:50) NFs with *φ* = 0.001–0.01% as thermal fluids in solar collectors was investigated by Fang and Xuan [90] for their thermo-optical properties and PTEC efficiency. A simulated sunlight with an irradiance of 1000 W/m^2^ was used while a self-designed device was engaged to evaluate the PTEC efficiency. As transmittance is inversely proportional to absorbance, increasing *φ* was noticed to improve absorbance, κ, and EC for all the tested samples. Peak absorption and κ were observed for CuO and CuO-ZnO (70:30) NFs, respectively. At the optical depth of 1 cm and *φ* = 0.01%, maximum SWEA efficiency and temperatures of 99.47% and 71.62 °C (CuO/DIW NF), 98.67% and 72.65 °C (CuO-ZnO (70:30)/DIW NF), and 94.78% and 71.81 °C (CuO-ZnO (50:50)/DIW NF) were obtained, respectively. Maximum PTEC efficiency of 97.4% (30 °C) and 34.7% (70 °C) was reported for CuO-ZnO (70:30)/DIW NF. Yu and Xuan [91] examined the influence of volume fraction (0.015–0.025%) and mixing ratio (7:3 and 8:2) on the absorbance and PTEC efficiency of CuO-Ag/DIW NFs engaged as thermal fluids in a DASC. A solar simulator was used to provide irradiance and the PTEC efficiency was calculated using an existing and applicable equation. They reported that the absorbance of the CuO-Ag/DIW NFs was higher than those of CuO/DIW NFs and DIW and it improved with volume fraction increase. The HNFs with a mixing ratio of 7:3 were noticed to produce higher absorbance, temperature change, and PTEC efficiency than those of 8:2. As the concentration of the HNFs (with a mixing ratio of 7:3) and solar irradiation (till 7000 s) increased, temperature change and PTEC efficiency improved. At a volume fraction of 0.025% and irradiation of 7000 s, the highest temperature change and PTEC efficiency of 34.1 °C and 96.11%, respectively, were recorded using CuO-Ag (7:3)/DIW NF as a thermal fluid.

Hjerrild et al. [92] experimented with the stability, thermal treatment, and optical properties of GL-based Ag-SiO_2_ NF as a liquid optical filter applied in PV/T collectors. The test samples were exposed to concentrated UV irradiation. They found that the HNF was stable under medium thermal treatment (125 °C) and accelerated high UV irradiation (300–1500 nm) exposure. The Ag-SiO_2_/GL NF was noticed to be applicable in a PV/T collector with high temperature and electrical output. Additionally, the Ag-SiO_2_/GL NF was observed to considerably enhance light transmission in comparison with Ag-SiO_2_/W NF coupled with its low price and wide range of temperatures. Zhou et al. [60] studied the use of GO-Au/DIW NFs at *φ* = 0.1–0.3 mg/mL as thermal media for steam generation. A solar simulator was used as the light source while a self-built device was used to estimate the PTEC efficiency. They showed that 0.2 mg/mL-GO-Au/DIW NF was the best thermal fluid with the highest evaporation rate, enhancement factor, and PTEC efficiency of 1.34 kg/m^2^ h, 2.35, and 84.1%, respectively. The observed results were strongly linked to 0.2 mg/mL-GO-Au/DIW NF possessing the highest absorption characteristics. HNFs of GO-Au/DIW were demonstrated to be better than the MNFs of Au/DIW and GO-DIW as working fluids for solar steam generation. The solar steam generation efficiency of GO-Au/DIW NF (0.2 mg/mL) was 20% higher than GO/DIW NF. Using the same light illumination, the temperature of Au-GO/DIW (0.2 mg/mL) NF was found to be 8–10 °C higher than DIW. The authors stressed the potential application of GO-Au/DIW NFs to include power generation, seawater desalination, and sterilization of waste.

Zeng and Xuan [93] studied the κ and PTEC effectiveness of DIW-based MWCNT-SiO_2_/Ag NFs (0.001–0.1%) with mixing ratios of 4:1–1:4 as operating fluids in volumetric solar collectors. To measure the PTEC efficiency, a simulative volumetric solar thermal conversion device was used with the test samples opened to a solar simulator as the light source. They showed that the SWEA fractions of DIW-based MWCNT-SiO_2_/Ag NFs ranged from 71.4% to 74.5% with mixing ratios of 4:1 and 1:4 having the highest and lowest values. However, 73.2% and 69.1% were obtained for DIW-based MWCNT and SiO_2_/Ag NFs. On exposure to irradiation for 1 h, temperatures of 48.1–59.3 °C, 47.7–56.6 °C, and 48.6–62.3 °C were recorded for MWCNT, SiO_2_/Ag, and MWCNT-SiO_2_/Ag (with volume fractions of 0.001–0.1%) NFs, respectively, and 46.9 °C for DIW. The HNFs attained maximum PTEC efficiency of 97.6% and 42.7% at 35 °C and 70 °C, respectively. This indicated that the HNFs have higher PTEC efficiency than MNFs and that the PTEC efficiency was reduced with temperature increase. The obtained results were strongly connected to the higher κ and absorbance values recorded for the HNFs in comparison with MNFs and DIW. Shi et al. [59] studied the PTEC and purification capability of Fe_3_O_4_-TiO_2_/DIW NF with *φ* = 0.1 g/L in solar energy applications. The experiment exposed the HNFs to a solar simulator and the PTEC was evaluated using an empirical equation. The results proved that with increasing solar power intensities (1–10 suns), the thermal receiver efficiency was observed to reduce while the degradation and evaporation (at a steady-state) efficiency slowly increased. At 1 sun, maximum thermal receiver efficiency of 76.4% and degradation efficiency of 85% were recorded. A magnetic field was used to recover the HNPs of the HNF for purification purposes and this led to recovery efficiency of 47.4% and 94.0% with a magnetic field intensity of 25 mT and 100 mT, respectively. After 1200 s, total material recovery was achieved under magnetic field influence and no change in the material was observed in the absence of the magnetic field.

The possible manipulation of the thermal, optical, and photothermal properties of various NFs (TiN, Fe_3_O_4_, and Fe_3_O_4_-TiN with volume fractions of 0.005–0.04%) under diverse magnetic field strengths and orientations were investigated by Zeng and Xuan [94]. A simulative volumetric solar thermal conversion set up was used to measure the photo-thermal property of HNFs. They demonstrated that transmittance decreased with an increase in *φ* whereas the opposite was observed for absorbance. A rise in the temperature of the studied samples was noticed as solar irradiation time and *φ* increased. With 1 h solar irradiation time and volume fraction of 0.005%, the SWEA fraction and temperature (of studied NFs) increment order of Fe_3_O_4_-TiN > Fe_3_O_4_ > TiN was observed. The parallel configuration of the incident light and magnetic field direction was noticed to produce better results than the perpendicular case, except for the absorbance where further reduction was recorded. Under magnetic field exposure, the SWEA fraction, and temperature of Fe_3_O_4_-TiN and Fe_3_O_4_ NFs were reduced with increasing magnetic field strength. The obtained findings (under magnetic field influence) were due to the improvement of their κ values and this revealed the potential alteration of absorbance, absorbed solar energy, PTEC performance, and heat transfer of magnetic HNFs for solar applications.

Qu et al. [95] examined the optical properties and PTEC performance of CuO (0.01–0.25 wt%)-MWCNT (0.005–0.0015 wt%)/DIW NFs. Light from a solar simulator was beamed on the test samples for the photothermal property of the samples. The results proved that the transmittance decreased as the concentration of the HNFs increased but the EC enhanced with it. Using DIW-based 0.15 wt% CuO + 0.005 wt% MWCNT NF and at an optical penetration of 1 cm and solar radiation time of 45 min, the SWEA fractions of HNF were 99.2%. The highest terminal temperature surge (14.1 °C) was reached at the optimum mixing ratio of the HNFs and irradiation time of 45 min, in comparison with DIW. The use of HNFs, especially at the optimum mixing ratio yielded working fluids with better PTEC performance than MNFs (in this case DIW-based CuO and MWCNT NFs). The stability, optical, and thermal properties, and PTEC efficacy of DIW-based rGO, rGO-Ag (15), and rGO-Ag (30) NFs at varying concentrations (10–100 mg/L) as thermal fluids in a DASC, were examined by Mehrali et al. [96]. A solar simulator was engaged as a light source in the experiment. An evaluation of the PTEC efficiency was carried out using an established empirical equation. The absorbance, EC, and SWEA fraction of the MNF and HNFs were noticed to improve with concentration while the transmittance decreased with concentration. With an irradiation time of 2000 s, the highest change in temperature on the surface (top) of the collector was 24 °C, 27.4 °C, and 28.6 °C for rGO, rGO-Ag (15), and rGO-Ag (30) NFs, respectively. In addition, PTEC efficiency of 63.3% (at 80 mg/L), 78% (at 100 mg/L), and 77% (at 40 mg/L) was achieved with rGO, rGO-Ag (30), and rGO-Ag (15) NFs, respectively, at 1 sun irradiation and 2000 s irradiation time. The best candidate for DASC application based on cost was rGO-Ag (15) NF with 40 mg/L concentration and 20 mm collector height.

The PTEC performance, SAR, and cost of the deployment of DIW-based Au, Cu, carbon black, and Au-Cu NFs as thermal fluids in DASC were examined by Zeiny et al. [97]. Light was beamed on the test samples using a sun simulator while the PTEC efficiency was estimated using an empirical equation. They observed that increasing the irradiation time slightly increased the temperature of the studied samples while an increase in mass concentration moderately enhanced the temperature of the samples. The PTEC efficiency and enhancement were enhanced as the mass concentration of the MNFs and HNF increased but they decreased as irradiation time increased. Additionally, the SAR and cost of the MNFs reduced and increased with concentration increase, respectively. With PTEC efficiency of 125%, 72%, and 100% for carbon black (100 mg/L), Au (150 mg/L), and Cu (3000 mg/L) NFs, respectively, the HNFs did not show an increase in this parameter. Based on SAR and cost values, the carbon black NF appeared to be the best MNF. Bhalla et al. [98] experimented the PTEC characteristics of DIW-based Al_2_O_3_ (20–150 mg/L) + Co_3_O_4_ (20–80 mg/L) NFs in DASCs using surface absorption and blended NF absorption systems. An artificial light source was used to simulate solar irradiation. The effectiveness of these systems was performed under similar working conditions. The results proved that the HNFs have an SWEA fraction of over 80% at a penetration depth of 20 mm. The addition of different mass fractions of Co_3_O_4_/DIW NFs to various fixed mass fractions of Al_2_O_3_/DIW NFs showed an increase in the SWEA fraction. At an optimum mass fraction of 40 mg/L Al_2_O_3_ + 40 mg/L Co_3_O_4_ NF, the peak temperature rise was attained with the HNFs as thermal media. Under similar working conditions, the blended NF absorption system was observed to yield a higher temperature (5.4 °C) than the surface absorption system due to the deployment of HNFs, therefore, making them good candidates for DASC.

Silicone oil-based ZnO-Au NFs with *φ* = 0.1–1.0 mg/mL were deployed to examine the optical properties and PTEC performance under varying irradiation duration and intensities [99]. The tested HNFs were subjected to simulated solar radiation. PTEC efficiency was estimated using an empirical equation. Results proved that the transmittance of the studied sample decreased with *φ* while the EC was enhanced with *φ*. Increasing solar radiation time, *φ*, and height (from the bottom of the beaker) improved the temperature of the studied samples at a stirring rate of 1000 rpm. On exposure of the silicone oil-based ZnO-Au NFs (*φ* = 1.0 mg/mL) to 10 kW/m^2^ solar radiation for less than 1 h, the temperature was raised to around 125 °C. The PTEC efficiency of 36%, 49%, and 60% was obtained for ZnO-Au/silicone oil NFs with concentrations of 0.1, 0.5, and 1 mg/mL, respectively. In comparison with silicone oil (17%), PTEC efficiency improvement of 240% was achieved using 1 mg/mL ZnO-Au/silicone oil NF. Increasing the solar radiation intensity was observed to enhance the temperature of 1 mg/mL ZnO-Au/silicone oil NF. They demonstrated that ZnO-Au/silicone oil NFs were effective working fluids for application in DASCs.

Using EG-based FeNi/C NFs with concentrations of 5–50 ppm, Wang et al. [100] investigated their optical properties and PTEC performance under forced convection flow conditions in the absence and presence of a rotating magnetic field (50 mT). The HNFs were exposed to simulated solar radiation as the PTEC efficiency was evaluated using an equation. Results revealed that an increase in concentration led to the enhancement of EC and SWEA fraction and a reduction in transmittance for the studied samples. At an optical depth of 1 cm, absorbed energy was observed to appreciate with an increase in concentration and irradiation time. With solar irradiation time of 3600 s, the FeNi/C-EG NFs recorded absorbed energy of 1024.9 J, 1088.3 J, 1036.4 J, and 1022.4 J (without magnetic field) and 1069.9 J, 1233.6 J, 1269.2 J, and 1254.8 J (with the magnetic field) at 5 ppm, 15 ppm, 25 ppm, and 50 ppm, respectively, in comparison with EG (872 J). In addition, PTEC efficiency of 47.4%, 50.4%, 47.9%, and 47.3% (without magnetic field) and 49.5%, 57.1%, 58.7%, and 58.1% (magnetic field) was obtained for FeNi/C-EG NFs at 5 ppm, 15 ppm, 25 ppm, and 50 ppm, respectively, as compared with EG (40.4%). The magnetic field manipulation of the magnetic FeNi/C-EG NFs as working fluids in a DASC appears to improve its PTEC efficiency. Gulzar et al. [101] investigated the doping of a high-temperature thermal fluid (therminol-55) with Al_2_O_3_, TiO_2_, and Al_2_O_3_-TiO_2_ NPs as working fluids for concentrated solar collectors. To estimate the photothermal energy conversion, the test samples were subjected to simulated solar radiation as a light source. The MNFs and HNFs were formulated at weight concentrations of 0.05–0.5 wt% and subsequently studied the increase in heat gain, temperature, and temperature enhancement. Results demonstrated that due to higher absorption, maximum PTEC (heat gain) was observed with the HNFs followed by Al_2_O_3_ and TiO_2_ NFs. Though TiO_2_ NFs yielded the highest absorbance, Al_2_O_3_-TiO_2_ and Al_2_O_3_ NFs showed maximum temperatures of 158.6 °C and 152.9 °C, respectively, compared with 149.6 °C for TiO_2_ NFs and 125.8 °C for therminol-55. With the same irradiation time of 5000 s, a peak temperature improvement of 34 °C was noticed with 0.5 wt% Al_2_O_3_-TiO_2_/therminol-55 NF. For both Al_2_O_3_ and TiO_2_ NFs, increasing the weight concentration was observed to increase the maximum temperature enhancement.

Jin et al. [102] examined the SWEA capacity and PTEC performance of different DIW-based MNFs (Cu, Au, and Fe_3_O_4_) and HNFs (Cu-Au, Fe_3_O_4_-Au, Fe_3_O_4_-Cu, and Fe_3_O_4_-Cu-Au with equal volume fractions) as working fluids in DASC. The tested HNFs were subjected to a simulated light source and the PTEC efficiency was estimated using an equation. They showed that with an irradiation time of 300 s, the temperatures of water-based Cu, Au, Fe_3_O_4_, Cu-Au, and Fe_3_O_4_-Au NFs were 30.36 °C, 30.89 °C, 29.74 °C, 31.2 °C, and 30.47 °C, respectively. Additionally, at 1.5 cm optical depth, the PTEC efficiency of Cu, Au, Fe_3_O_4_, Cu-Au, Fe_3_O_4_-Au, Fe_3_O_4_-Cu, and Fe_3_O_4_-Cu-Au NFs was 75.4%, 76.2%, 61.2%, 80.2%, 70.7%, 76.9%, and 75.5%, respectively. It was observed that the PTEC efficiency of the HNFs enhanced as *φ* increased with optimum values achieved using Cu-Au (Au–0.52 volume fraction) and Fe_3_O_4_-Cu (Cu–0.46 volume fraction) NFs. Using DIW-based SiO_2_/Ag-CuO NF as a working fluid in a DASC, Joseph et al. [103] examined the thermo-optical properties and PTEC performance. Tested samples of HNFs were exposed to sunlight and the PTEC was calculated using an existing equation. The formulation of a stable SiO_2_/Ag-CuO NF was optimized via the mass fractions of CuO NPs, SiO_2_/Ag NPs, SDS (as a surfactant), relative κ, and SWEA fraction. Their results showed optimal values of 864.7 mg/L for CuO NPs, 206.3 mg/L for SiO_2_/Ag NPs, and 1996.2 mg/L for SDS to produce good relative κ (1.234) and SWEA fraction (82.8%). Using the HNF, a peak temperature of 45.7 °C was recorded against 38.8 °C for DIW. Additionally, the SiO_2_/Ag-CuO NF absorbed maximum energy of 1942.6 J while that of DIW was 1239 J. They proposed a mathematical model for the estimation of relative κ and SWEA fraction as dependent on mass fractions of CuO, SDS, and SiO_2_/Ag.

**Table 2:** Photothermal performance of HNFs with different HNPs, mixing ratios, optical properties, surfactants, process methods, and stability tests.

References	HNF (Mix)/Base Fluid	*φ*	Optical	Nano-Size (nm)	Stability	Result
Tong et al. [61]	MWCNT-Fe_3_O_4_ (20:80–80:20)/EG-W (20:80)	0.01 wt% (25°)	Absorbance, transmittance, and κ	MW-10–20 Fe_3_O_4_-10	UV, ZP, and Visual (2-step)	SWEA fraction and PTEC efficiency of the HNFs were higher than the Fe_3_O_4_ NF.
Gulzar et al. [101]	Al_2_O_3_-TiO_2_ (60:40)/Therminol-55	0.05–0.5 wt%	Absorbance and transmittance	Al_2_O_3_-<80 TiO_2_-15–25	Visual (72-D), UV, Oleic (2-step)	At the same irradiation time of 5000 s, the highest temperature improvement (34 °C) was noticed with 0.5 wt% Al_2_O_3_-TiO_2_/therminol-55 NF.
Zhou et al. [60]	GO-Au/DW	0.1–0.3 mg/mL	Absorbance and transmittance	-	UV, ZP, glucose-functionalized (2-step)	The 0.2 mg/mL-GO-Au/DIW NF was the best thermal fluid with peak evaporation rate, enhancement factor, and PTEC efficiency of 1.34 kg/m^2^ h, 2.35, and 84.1%, respectively.
Hjerrild et al. [92]	Ag-SiO_2_/GL		Absorbance and transmittance	-	UV	Ag-SiO_2_/GL NF was noticed to be stable under medium-temperature thermal treatment and accelerated high UV irradiation exposure. Ag-SiO_2_/GL NF is better than Ag-SiO_2_/W NF in a PV/T collector with high temperature and electrical output.
Shi et al. [59]	Fe_3_O_4_-TiO_2_/DIW		Absorbance and transmittance	Fe_3_O_4_-TiO_2_-50	UV	The highest thermal receiver efficiency of 76.4% and degradation efficiency of 85% were recorded at 1 sun. Increasing magnetic field intensity enhanced degradation efficiency from 47% (25 mT) to 94% (100 mT).
Zeng and Xuan [93]	MWCNT-SiO_2_/Ag (4:1–1:4)/DIW	0.001–0.1%	Absorbance, transmittance, and κ	MWCNT-8–15	UV, ZP, CTAB	Maximum PTEC efficiency of 97.6% was achieved using HNFs, making them better thermal fluids than MNFs. This was due to the high κ and absorbance values of HNFs.
Bhalla et al. [98]	Al_2_O_3_-Co_3_O_4_/DIW	Al_2_O_3_ (20–150 mg/L) Co_3_O_4_ (20–80 mg/L)	Absorbance and transmittance	Al_2_O_3_-13 Co_3_O_4_-10–30	Triton X-100 (2-step)	The optimum mass fraction of 40 mg/L Al_2_O_3_ + 40 mg/L Co_3_O_4_ NF yielded the highest temperature rise. Under similar working conditions, the blended NF absorption system was noticed to yield a higher temperature (5.4 °C) than the surface absorption system.
Li et al. [104]	SiC-MWCNT (8:2)/EG	0.01–1 wt%	Absorbance and transmittance	SiC-40 MWCNT-20	ZP, UV, PVP, (2-step)	The SWEA fraction of 0.5 wt% SiC-MWCNT/EG NF was 99.9% at a penetration distance of 1 cm. With an irradiation time of 10 min, the peak PTEC efficiency was 97.3% using SiC-MWCNT/EG NF with *φ* = 1 wt%, which was 48.6% more than that of EG.
Jin et al. [102]	Cu-Au, Fe_3_O_4_-Au, Fe_3_O_4_-Cu (1:1), and Fe_3_O_4_-Cu-Au (1:1:1)/DIW	0.06–1 vol%	Absorbance and transmittance	Cu-60–80	UV	The PTEC efficiency of Cu, Au, Fe_3_O_4_, Cu-Au, Fe_3_O_4_-Au, Fe_3_O_4_-Cu, and Fe_3_O_4_-Cu-Au NFs was 75.4%, 76.2%, 61.2%, 80.2%, 70.7%, 76.9%, and 75.5%, respectively, at 1.5 cm optical depth.
Qu et al. [95]	CuO-MWCNT/DIW	0.0015 wt% and 0.005 wt% (MWCNT), 0.01–0.25 wt% (CuO)	Extinction coefficient, absorbance, and transmittance.	MWCNT->50	UV	Using DIW-based 0.15 wt% CuO + 0.005 wt% MWCNT NF and at an optical distance of 1 cm and irradiation time of 45 min, the SWEA fractions of HNF was 99.2%. The HNFs have improved PTEC efficiency better MNFs.
Mehrali et al. [96]	rGO-Ag/DIW	10–100 mg/L	Extinction coefficient, absorbance, transmittance, κ, and μ.	Ag-25–45	UV	The PTEC efficiency of 63.3% (80 mg/L), 78% (100 mg/L), and 77% (40 mg/L) was achieved with rGO, rGO-Ag (30), and rGO-Ag (15) NFs, respectively, at 1 sun irradiation intensity and 2000 s irradiation time. The rGO-Ag (15) NF was the best thermal fluid at a collector height of 2 cm.
Campus et al. [105]	Au, Ags, Agc, Cu, GOh, GOl, and Ag-GOl/water	40 and 100 mg/L	Extinction coefficient, absorbance, transmittance, and κ	Au-20, Ags-60, Agc-40–120, Cu-10–100, and Ag-Gol-18	UV	Under natural solar irradiation (high flux) of 600 s, a higher influence of the NPs shapes on the temperature difference and PTEC efficiency for NFs and HNFs was observed in comparison with artificial irradiation of 1 sun for 3000 s.
Kimpton et al. [62]	Ag, SiO_2_, and Ag-SiO_2_/W	-	Absorbance and optical density	-	UV (1-step)	The highest temperature and enhancement of 44.1 °C and 102% and 41.7 °C and 91% were observed for Ag and Ag-SiO2 NFs in comparison with water (21.8 °C), respectively. The PTEC efficiency of Ag-SiO_2_ and Ag NFs was around three-fold more than that of SiO_2_ NF.
Joseph et al. [103]	SiO_2_/Ag-CuO/DIW	-	κ	CuO-<50	UV, ZP, SDS (2-step)	Optimal values of 206.3 mg/L, 864.7 mg/L, and 1996.2 mg/L for SiO_2_/Ag, CuO, and SDS produced good relative thermal conductivity (1.234) and SWEA fraction (82.8%). With the HNF, a peak temperature of 45.7 °C was recorded against 38.8 °C for DIW.
Zeiny et al. [97]	Au-Cu (1:1)/DIW		Absorbance	-	UV, DLS, ZP,	With PTEC efficiency of 125%, 72%, and 100% for carbon black (100 mg/L), Au (150 mg/L), and Cu (3000 mg/L) NFs, respectively, the HNFs showed no increase in this variable. Subject to SAR and cost results, the carbon black NF was a suitable thermal fluid.
Wang et al. [100]	FeNi/C (2.19:2.41:95.4)/EG	5–50 mg/L	Extinction coefficient, absorbance, transmittance, and B = 50 mT.	FeNi/C-8-10	UV, Visual (2-step)	With solar irradiation time of 3600 s, PTEC efficiency of 47.3–50.4% (without magnetic field) and 49.5–58.7% (magnetic field) for EG-based FeNi/C NFs at 5–50 ppm, as compared with EG (40.4%).
Zhu et al. [106]	Ag-Au-ZNG, Au-ZNG, Ag-ZNG/EG	10–100 ppm	Extinction coefficient, absorbance, and transmittance.	-	UV (2-step)	At an optical depth of 1 cm, concentration of 100 ppm, and solar irradiation of 3000 s, maximum temperature rise, SWEA fraction, and PTEC efficiency of 58.6 °C, 97.1%, and 74.35% were obtained for Ag-Au/ZNGs NF, respectively.
He et al. [107]	Ag-TiO_2_/EG-W	50–200 ppm	Extinction coefficient, absorbance, and transmittance.	Ag-TiO_2_-23.6 TiO_2_-2	UV	The PTEC efficiency of Ag-TiO_2_ NF (at 200 ppm) and EG-W (60:40) was 39.9% and 78.1%, respectively, while the PV efficiency was 5.6% for Ag-TiO_2_ NF. The overall PTEC efficiency of Ag-TiO_2_ NF was 83.7% (200 ppm) whereas 54.1% was recorded for EG-W (60:40).
Wang et al. [99]	ZnO-Au/silicone oil	0.1–1 mg/mL	Extinction coefficient, absorbance, transmittance, and c_p_.	Au-13.3, ZnO-0.08 μm	UV (2-step)	The PTEC efficiency of 36%, 49%, and 60% was obtained for ZnO-Au/silicone oil NFs with concentrations of 0.1, 0.5, and 1 mg/mL, respectively. PTEC efficiency improvement of 240% was attained with 1 mg/mL ZnO-Au/silicone oil NF.
Chen et al. [86]	Au-Ag/DIW	Au (0.5–2.5 ppm) + Ag (0.15 ppm and 0.5 ppm)	Absorbance.	Au-10 Ag-30	UV	The PTEC efficiency for Au (1.75 ppm) + Ag (0.15 ppm), Au (1.75 ppm), and Ag (0.15 ppm) NFs was 30.97%, 19.01%, and 11.90% was obtained respectively.
Zeng and Xuan [94]	Fe_3_O_4_-TIN/DIW	0.005–0.04%	Extinction coefficient, absorbance, magnetization, and transmittance.	Fe_3_O_4_-100 TIN-15	UV, Visual	With 1 h solar irradiation and volume fraction of 0.005%, the SWEA fraction and temperature of NFs increased in the order of Fe_3_O_4_-TiN > Fe_3_O_4_ > TiN. The parallel orientation of incident light and magnetic field direction was noticed to produce better results than the perpendicular case, except for the absorbance.
Carrillo-Torres et al. [87]	Au-Ag	-	-	-	DLS	For the HNF, maximum photothermal efficiency of 74.68% was obtained while a temperature of 20 °C was recorded after exposing the sample to 15 min of irradiation.
Shende and Sundara, [108]	rGO-MWCNT/DIW and EG	-	κ	-	UV, PEG: SLS (2:1),	The thermal and optical properties of rGO-MWNT NF were observed to be enhanced compared with DIW and EG.
Chen et al. [88]	CuO-ATO (1:9–9:1)/DIW	0.02–0.12 vol%	-	-	UV, ZP, pH, sodium citrate (2-step)	Maximum SWEA fraction, temperature change, and PTEC efficiency of 99.6%, 43.6 °C, and 92.5%; 89.5%, 39.8 °C, and 81.3%; and 89.8%, 39.6 °C, and 80.7% were recorded for CuO-ATO, CuO, and ATO NFs, respectively.
Xuan et al. [64]	TiO_2_-Ag/DIW	0.002–0.15%	-	TiO_2_-30 Ag-20	(2-step)	Absorbed energy, temperature, and thermal efficiency of 57.89, 390.88, and 413.36 W/m^2^; 60.21 °C, 66.65 °C, and 66.93 °C; and 16.07%, 20.86%, and 20.9% were obtained for TiO_2_, Ag, and TiO_2_-Ag NFs, respectively.
Shin et al. [77]	MWCNT-Fe_3_O_4_ (1:1)/EG-W (20:80 wt%)	0.005–0.2 wt%	Transmittance, B = 250–750 G, and κ	-	Visual (2-step)	The temperature and PTEC efficiency of 0.2 wt% MWCNT-Fe_3_O_4_ NF was 45 °C and 32% (without magnetic field) and 60 °C and 45% (with the magnetic field of 750 G). Under 750 G magnetic intensity, the total stored energy of 0.2 wt% MWCNT/Fe_3_O_4_ NF was enhanced by 61.5%.
Li et al. [104]	SiC-MWCNT (80:20)/EG	0.01–1 wt%	Extinction coefficient, absorbance, and transmittance.	SiC- 40 MWCNT- 20	ZP, UV, Visual, PVP-K30, (2-step)	At a maximum SWEA fraction of over 99.9%, the temperature difference of close to 110 °C, and PTEC efficiency of 97.3% were obtained with 1 wt% SiC-MWCNT/EG NF.

Zhu et al. [106] examined the utilization of EG-based ZNGs, Au/, Ag/, and Ag-Au/ZNGs NFs (with concentrations of 10–100 ppm) as working fluids in DASC systems. With the use of a laboratory-built device, the PTEC efficiency was evaluated. A solar simulator was utilized as a source of light beamed on the tested samples. The optical properties and the PTEC capacity of the MNFs and HNFS were studied. With an increase in the concentration of the MNFs and HNFs, the SWEA fraction and EC were enhanced while the transmittance was reduced. This led to the improvement of PTEC efficiency with a rise in temperature, irradiation time, and concentration for all the studied samples due to the plasmonic effect and hybridization of HNPs. At an optical depth of 1 cm, a concentration of 100 ppm, and solar irradiation of 3000 s, SWEA fraction and PTEC efficiencies of 90.1%, 94.9%, 95.4%, and 97.1% and 69.25%, 70.35%, 72.41%, and 74.35% were obtained for ZNGs, Ag/, Au/ and Ag-Au/ZNGs NFs, respectively. The temperature of Ag/, Au/, and Ag-Au/ZNGs NFs were more than that of ZNGs, with Au/ZNGs NFs having the highest temperature (58.61 °C).

For a PV/T system, He et al. [107] examined the SWEA and PTEC performance of EG-W (60:40)-based Ag-TiO_2_ NFs with concentrations of 50–200 ppm as beam-splitter in a temperate region. A theoretical model based on energy balance was used to evaluate PTEC efficiency. A solar simulator was deployed as a light source to illuminate the test samples. The transmittance and absorbance were inversely and directly proportional to the concentration of the HNFs. After 35 min of exposure to solar radiation, the temperature of Ag-TiO_2_ NFs (200 ppm) was increased to 16.6 K. The current and power density were noticed to improve as voltage decreased and increased, respectively, while a reduction in concentration enhanced the current and power density of all the samples. The PTEC efficiency of Ag-TiO_2_ NF (at 200 ppm) and EG-W (60:40) was 39.9% and 78.1%, respectively, while the PV efficiency was 5.6% for Ag-TiO_2_ NF (at 200 ppm). The overall PTEC efficiency of Ag-TiO_2_ NF was 70.7%, 74.8%, and 83.7% at concentrations of 50, 100, and 200 ppm whereas 54.1% was recorded for EG-W (60:40). With higher merit functions of 1.89 (50 ppm), 1.91 (100 ppm), and 2.04 (200 ppm) for the HNF-based splitters compared with 1.64 for the base fluid, coupled with worth factor of 3 for Ag-TiO_2_ NF (at 200 ppm), the HNF-based splitter at the highest concentration appeared suitable for PV/T applications.

Campus et al. [105] studied the thermal and optical properties, and PTEC performance of water-based spherical (Au, Cu, and Ags), non-spherical (Agc, GOh, and GOl), and hybrid (GOl-Ag) NFs as working fluids in DASC. The influence of particle types and shapes, natural and artificial irradiation, irradiation time (600 s and 3000 s), and concentration (40 mg/L and 100 mg/L) on the PTEC efficiency were studied. They noticed that SWEA efficiency increased with a decrease in temperature while the temperature change increased with irradiation time. With 1 sun and irradiation of 3000 s, an order of GOl-Ag NF (91%) > GOl NF (73%) > GOh NF > Agc NF (71%) > Au NF (65%) > Cu NF (60%) > Ags NF (40%) was observed for the SWEA efficiency under different concentrations of 40 mg/L and 100 mg/L. The subjection of the MNFs and HNFs to natural solar irradiation (high flux) for 600 s resulted in a higher influence of the NPs shapes on the temperature difference and SWEA efficiency in comparison with artificial irradiation of 1 sun for 3000 s. This study showed that the GOl-Ag and Agc NFs were the most suitable thermal fluids as they have the highest SWEA efficiency. Recently, Tong et al. [61] examined the SWEA and transmittance characteristics and the PTEC efficiency of MWCNT-Fe_3_O_4_/W-EG (80:20) NFs at varying mixing ratios (80:20–20:80). Radiation of the sun was used as a light source and the PTEC efficiency was estimated using established equation. They showed that increasing the ratio of the MWCNT particle in the HNF caused an enhancement of the SWEA fraction and PTEC efficiency and a reduction in the transmittance characteristics. The highest transmittance (29%) was recorded for the HNF with a mixing ratio of 80:20 while 89% was observed for Fe_3_O_4_/W-EG (80:20) NF, all at 0.01 wt%. Thus, indicating that the MNF has a better transmittance property than the HNF. With higher thermal and optical properties of MWCNT-Fe_3_O_4_/W-EG NFs compared with Fe_3_O_4_/W-EG NFs, higher SWEA fraction (1–maximum), and PTEC efficiency were observed.

For the first time, an attempt was made by Kimpton et al. [62] to investigate the optical and stability properties and PTEC efficiency of water-based Ag, Ag-SiO_2_, and SiO_2_ NFs under natural and simulated solar exposure. Simulated sunlight was deployed as a light source in the experiment while the PTEC efficiency was estimated using the applicable existing equation. Results demonstrated the instability of Ag-SiO_2_ and Ag NFs on exposure to natural solar irradiation, with a higher tendency observed for Ag NF. With all the studied samples and under simulated solar irradiation, the temperature change rose as irradiation time increased. The highest temperature was observed with Ag NF (44.1 °C), followed by Ag-SiO_2_ NF (41.7 °C) and SiO_2_ (23.6 °C), with corresponding enhancements of 102%, 91%, and 8% in comparison with water (21.8 °C), respectively. The PTEC efficiency of Ag-SiO_2_ and Ag NFs was around three-fold more than that of SiO_2_ NF. The stability, optical properties, SWEA, and PTEC performance of therminol^®^66-based GO-MWCNT NFs (with *φ* = 10–150 ppm) as potential working fluids in a DASC under indoor and outdoor conditions were studied by Qu et al. [109]. Tested samples were subjected to simulated and real sunlight and the evaluation of the PTEC efficiency was calculated using an empirical equation. Similar to other studies, increasing *φ* enhanced EC and SWEA fraction and reduced the transmittance of the studied samples. Maximum temperature and PTEC efficiency were accomplished with HNF at an optimum concentration of 100 ppm. At an optical depth of 1.75 cm, a concentration of 150 ppm, and a solar radiation time of 45 min, 99% SWEA capacity was recorded. Under indoor and outdoor conditions, the temperature of 100 ppm-GO-MWCNT/therminol^®^66 NF was 94 °C and 153 °C and 11.6 °C and 97 °C higher than therminol^®^66, respectively.

The thermal-optical properties and the PTEC performance of EG-W (20:80)-based MWCNT-Fe_3_O_4_ (1:1) NFs with *φ* = 0.005–0.2 wt% as working fluids in a solar collector were studied by Shin et al. [77] under varying magnetic fields (250–750 G). Irradiation of the tested samples was carried out using solar simulation. Estimation of the PTEC efficiency was done using the established equation. Increasing the magnetic field was observed to enhance κ and temperature, and it reduced the PTEC efficiency of the HNFs. As the *φ* increased, κ, SWEA fraction, temperature, PTEC efficiency, and total stored energy were enhanced. On exposure to solar radiation of 560 W/m^2^ for 1 h and at an optical length of 1 cm, the temperature and PTEC efficiency of 0.2 wt% MWCNT-Fe_3_O_4_ NF were 45 °C and 32% (without magnetic field) and 60 °C and 45% (with a magnetic field of 750 G), respectively. On exposure of 0.2 wt% MWCNT-Fe_3_O_4_ NF to 20 min of solar radiation, the PTEC efficiency was 65% and 54% in the presence and absence of magnetic field intensity of 750 G, respectively. Under 750 G magnetic intensity, a peak total stored energy of 15 MJ was recorded for 0.2 wt% MWCNT/Fe_3_O_4_ NF, which translated to a 61.5% increment compared with the case of no magnetic field. This study showed an order of MWCNT > MWCNT-Fe_3_O_4_ > Fe_3_O_4_ for the PTEC efficiency and temperature due to the κ, transmittance, and SWEA efficiency of these MNFs and HNF. Li et al. [104] explored the stability, optical properties (EC and transmittance), and PTEC efficiency of SiC-MWCNT (8:2)/EG NFs with *φ* = 0.01–1 wt% as efficient working fluids in DASC. Tested HNFs were exposed to solar simulated light for the photothermal experiment, while the PTEC efficiency was evaluated using the established equation. The results revealed excellent stability and SWEA characteristics (200–1100 nm) for SiC-MWCNT/EG NFs as working fluids. Increasing the mass fraction of SiC-MWCNT/EG NF was noticed to enhance the EC, relative absorbance, solar irradiation, temperature difference, irradiation time, and PTEC performance but it reduced with transmittance. At an optical depth of 1 cm, the SWEA fraction of SiC-MWCNT/EG NF with *φ* = 0.5 wt% was 99.9%. The highest temperature difference for the HNFs was 32 °C above that of EG. With an irradiation time of 10 min, the peak PTEC efficiency (97.3%) was achieved using SiC-MWCNT/EG NF with *φ* = 1 wt%, which was 48.6% more than the value recorded when EG was used. The authors recommended the utilization of 0.5 wt% and 1 wt% SiC-MWCNT/EG NFs in DASC due to the PTEC performance.

Factors such as nano-size [88], types, shapes of NPs, concentration, Brownian motion, nano-layering, stability, optical properties (absorptivity and EC) and κ of HNPs, mixing ratios, magnetic field strength and orientation [77], types of base fluids have been shown to significantly influence the SWEA fractions, HNPs temperature rise [93], and consequently the PTEC efficiency of HNFs [94] as working fluids in solar energy application. Besides stability, nano-layering and Brownian motion are the two principal parameters that influence the thermal behavior of HNFs [101]. The solar-thermal properties of HNFs depend to a great extent on the relationship between the temperature change of HNFs and solar radiation time [104].

## 3. Solar Energy Application of Hybrid Nanofluids

The radiation of the sun releases solar energy which can be converted into heat and electricity. Outside of using photovoltaic cells to directly generate electricity from solar radiation, it is also practicable to harvest the same using steam generation, electric power generation, and thermal storage power technologies [95]. Solar collectors are engaged to absorb solar energy using different working fluids and the performance of the collectors is strongly connected to the optical and thermal properties of the working fluids. These optical properties include EC, absorptivity, transmissivity, scattering coefficient, SWEA, etc. [110]. PTEC is the most efficient of the solar energy harvesting methods of photothermal conversion, photochemical conversion, and photo-voltaic conversion [103]. The efficiency of solar collectors strongly depends on the thermophysical and optical properties of the HNFs used as working fluids, which are directly related to their PTEC performance [111]. The thermal properties, stability tests, and performances of various HNFs as working fluids for solar energy applications are presented in Table 3. Different HNPs (at varying mixing ratios and nano-sizes) and base fluids used to formulate the studied HNFs, and the characteristics of the various solar collectors used, are also included in Table 3. In addition, a summary of the key findings from the reviewed papers is presented in the table. Noteworthy works published between the inception of HNF studies and 2022 are reviewed in the compilation of Table 3.

**Figure 9:**
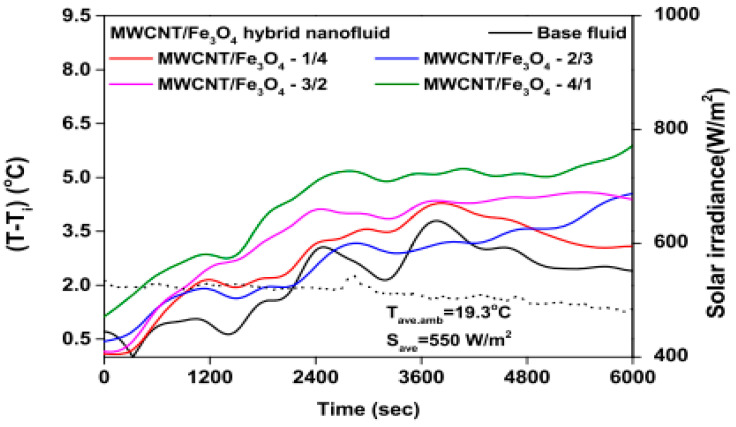
Temperature parameter and solar irradiance against increasing solar exposure duration for MWCNT/Fe_3_O_4_ nanofluids and base fluid (Adapted from Tong et al. [61]).

**Figure 10:**
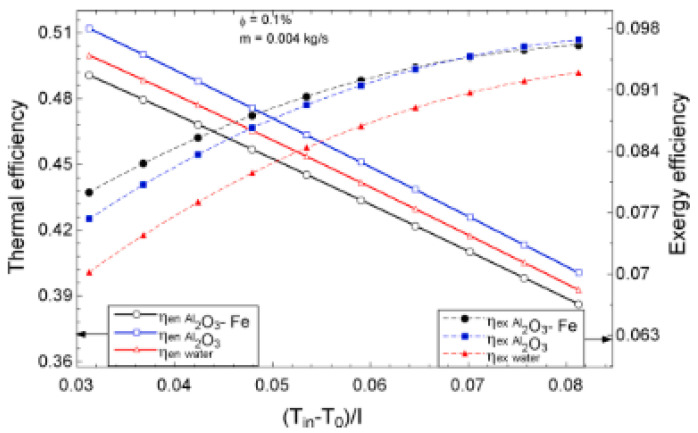
Thermal and exergetic efficiency performance of Al_2_O_3_-Fe nanofluid against temperature parameter for a flat plate collector application (Adapted from Okonkwo et al. [112]).

**Figure 11:**
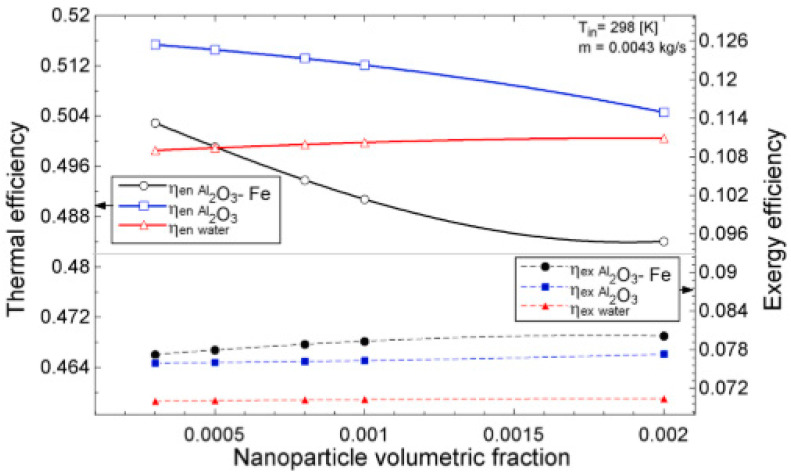
Thermal and exergetic efficiency performance of Al_2_O_3_-Fe and Al_2_O_3_ nanofluid and water against increasing volumetric fraction for a flat plate collector application (Adapted from Okonkwo et al. [112]).

**Figure 12:**
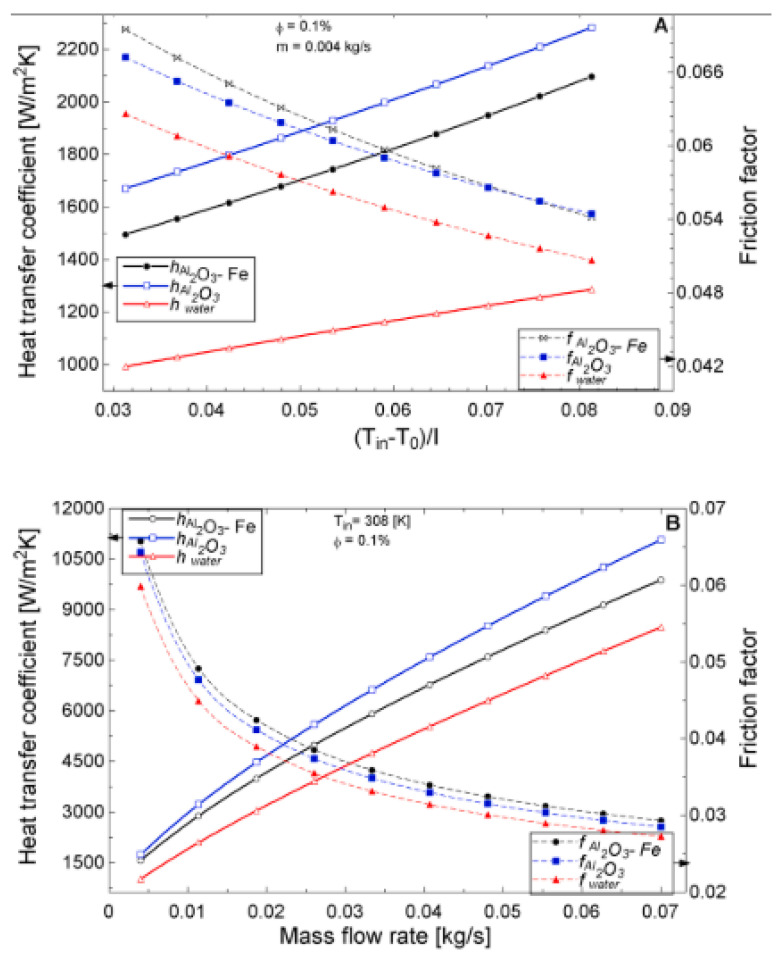
Coefficient of heat transfer coefficient and friction factor of 0.1% Al_2_O_3_-Fe and 0.1% Al_2_O_3_ nanofluid and water against varying mass flow rates at an inlet temperature of 308 K for a flat plate collector application (Adapted from Okonkwo et al. [112]). Heat transfer coefficient and friction factor performance as a function of (**A**) temperature (**B**) mass flow rate.

**Figure 13:**
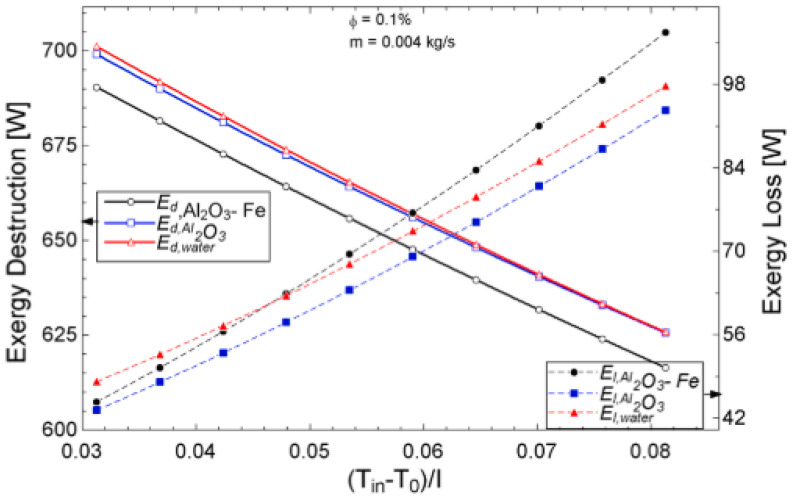
Exergy destruction and exergy loss performance of Al_2_O_3_-Fe and Al_2_O_3_ nanofluid and water against increasing temperature parameters for a flat plate collector application (Adapted from Okonkwo et al. [112]).

The temperature parameter and solar irradiance of HNFs of MWCNT-Fe_3_O_4_ (with varying mixing ratios) against increasing time of solar exposure are presented in Figure 9. Both solar irradiance and temperature parameter were improved as the exposure time increased with increasing amounts of MWCNT particles in the test samples found to cause this improvement. The energetic and exergetic efficiency of HNFs (MWCNT-Fe_3_O_4_) under varying temperatures and volume fractions as working fluids in an FPSC are provided in Figure 10 and Figure 11, respectively. Increasing temperature parameter was observed to reduce energetic efficiency and enhance exergetic efficiency. In Figure 12, the coefficient of heat transfer and friction factor of MWCNT-Fe_3_O_4_ nanofluids in an FPSC as functions of temperature parameters and mass flow rate is presented respectively while the exergy destruction and exergy loss of hybrid nanofluids in a solar collector under changing temperature parameters is illustrated in Figure 13. Further details of the study from which Figure 10, Figure 11, Figure 12 and Figure 13 are adapted are provided in paragraph three of Section 3.2.

### 3.1. Direct Absorption Solar Collectors

The influence of flow rate (20–100 cc/min) and mixing ratio (1:0–0:1) of DIW-based CeO_2_-CuO NFs (with *φ* = 0.1 vol%) on the thermal performance of a DASC was experimented in an outdoor and indoor environment under constant irradiation by Mohan and Sajeeb [116]. They showed that the thermal efficiency of the studied samples was enhanced as the flow rate increased while the same was improved as the mixing ratio of CuO NPs in the HNFs increased from 0.5 to 1.0 and as the CeO_2_ mixing ratio decreased from 1 to 0. In comparison with DIW and at a flow rate of 100 cc/min, the thermal efficiency of 13.8%, 18.1%, 24.3%, 24.9%, and 26.1% was obtained for the HNFs with mixing ratios of 1:0, 1:0.5, 1:1, 0.5:1, and 0:1, respectively. With the CeO_2_-CuO (1:1)/DIW NF, increasing the flow rate (20 to 100 cc/min) enhanced thermal efficiency by 16.5–51.5%. Additionally, at a flow rate of 100 cc/min, the thermal efficiency of CeO_2_-CuO/DIW NF was enhanced from 45.5% to 51.5%, when the mixing ratio of the HNF changed from 1:0 to 0.5:1. It was noticed that under indoor conditions, the thermal efficiency of all samples was improved as the flow rate increased with DIW-based CuO NF recording 35.4% thermal efficiency as the flow rate increased from 20 cc/min to 100 cc/min. It was observed that for the mixing ratios of 0:1, 1:1, and 0.5:1, the efficiency was almost the same.

Hong et al. [115] experimentally explored the efficiencies and solar vapor generation rates of HNFs (rGO + Ag, GO + Ag, rGO-Ag, and GO-Ag) with varying concentrations (0.113–1 mg/mL) under different light intensities (1–4 suns) as working fluids in different DASCs. The results showed that water mass loss increased as illumination time and concentration increased. Additionally, the relative efficiency and evaporation rate were observed to enhance with concentration increase. An order of rGO + Ag NFs > GO + Ag NFs > rGO-Ag NFs > GO-Ag NFs was noticed for the water mass loss, evaporation rate, and relative efficiency. At 3 suns, the relative efficiency ranges of 73.2–91.6%, 60.2–86.3%, 64.3–85.1%, and 54.1–79.9% were obtained for the GO + Ag, rGO + Ag, rGO-Ag, and GO-Ag NFs with concentrations of 1, 0.45, 0.225, and 0.1125 mg/mL, respectively. The elevated absorbance and plasmonic influence of the HNPs and high κ of graphene nanosheets were responsible for the obtained results. The future utilizations of rGO + Ag NF in applications such as desalination, power generation, and water treatment were recommended. Sreekumar et al. [63] investigated the thermal and exergy analysis of the deployment of DIW-ATO/Ag NF as a working fluid in a PTDASC. To formulate stable ATO-Ag/DIW NF, the mass fraction, surfactant mass fraction, and SWEA fraction were optimized. They reported an optimum mass fraction of 0.1% for ATO/Ag NF and surfactant with the corresponding SWEA of 90.1%. The results also demonstrated the enhancement of collector and exergy efficiency with an increase in temperature difference, irradiation intensity, and mass flow rate. At a mass flow rate of 0.022 kg/s and using the HNF, peak thermal efficiency due to a temperature surge of 12.6 °C was 63.5% while the highest exergy efficiency as a result of temperature difference of 8°C was 5.6%. Optical efficiency was noticed to improve as the angle of incidence decreased with a maximum value of 75% at 0°. Increasing the radiation penetration depth depreciated the transmittance of ATO-Ag/DIW NF while a rise in mass fraction enhanced its EC. This implied that the absorption of solar radiation can be achieved by either increasing the radiation penetration depth or mass fraction of the HNF. The authors proposed a mathematical model for the estimation of κ and SWEA fraction as dependent on mass fractions of ATO-Ag and SDS.

The influence of volume flow rate (1–5 L/h) and mixing ratio (0:100–100:0) of different HNFs (DW-based Al_2_O_3_-fly ash and SiO_2_-fly ash with *φ* = 2 vol%) employed as working fluids in a microchannel-based DASC on the energy and exergy performance was carried out by Thakur et al. [120]. The authors reported that increasing the volume flow rate enhanced thermal efficiency, pumping power, PEC, EGR, and exergy efficiency. The collector thermal and exergy efficiency was 72.82% and 59.23% and 73% and 68.09% for Al_2_O_3_-fly ash (80:20) and SiO_2_-fly ash (80:20) NFs, respectively. Higher pumping power of 30% and 33% than DW was obtained for Al_2_O_3_-fly ash (80:20) and SiO_2_-fly ash (80:20) NFs, respectively. In addition, at low flow rates, the Al_2_O_3_-fly ash (80:20) NF possessed a higher PEC (3.5) than SiO_2_-fly ash (80:20) NF with a PEC of 3.08. The Al_2_O_3_-fly ash (80:20) NF was observed to be a better working fluid compared with SiO_2_-fly ash (80:20) NF. This was because of the improved thermophysical properties of Al_2_O_3_-fly ash (80:20) NF relative to SiO_2_-fly ash (80:20) NF.

The collector performance of therminol^®^66-based GO-MWCNT NFs (with *φ* = 10–150 ppm) as potential working fluids in a DASC under indoor and outdoor conditions was studied by Qu et al. [109]. Under these conditions, the temperature of 100 ppm-GO-MWCNT/therminol^®^66 NF was 94 °C (indoor) and 153 °C (outdoor) compared with those of therminol^®^66 (11.6 °C and 97 °C). At irradiation times of 5 min and 45 min (under outdoor conditions), the collector efficiency using 100 ppm-GO-MWCNT/therminol^®^66 NF was 97% and 70%, respectively. The thermal stability of GO-MWCNT/therminol^®^66 NFs as determined prior to and after the experiments coupled with the obtained results emphasized the potential application of these thermal fluids for low-to-medium temperature in a DASC.

### 3.2. Flat Plate Solar Collectors

The influence of varying *φ* (0.5–2%) and thermal properties (*ρ*, κ, and μ) on the thermal efficiency of EG-W (75:25 wt%)-based Al_2_O_3_-CuO (70:30) NFs deployed as working fluids in an FPSC was investigated by Tahat and Benim [122]. They reported that a rise in the volume fraction led to the improvement of μ, *ρ*, κ, and thermal efficiency in comparison with water. Collector efficiency of 42–52% was obtained as the volume fraction increased from 0.5% to 2% when compared with water. The average improvement of the thermal efficiency for the FPSC relative to water was 45%. The improvement of h as κ increased for the HNFs resulted in thermal efficiency enhancement of the FPSC. Verma et al. [113] examined the effect of mass flow rate (0.01–0.05 kg/s), *φ* (0.25–2 vol%), solar intensity (380–1200 W/m^2^), and temperature parameter (0.0075–0.035) on the energetic and exergetic performance of MNFs (CuO/, MgO/, and MWCNT/DIW) and HNFs (MgO-MWCNT (80:20)/DIW and CuO-MWCNT (80:20)/DIW) in an FPSC. Results revealed that optimum values of 0.75–0.8 vol%, 800–900 W/m^2^, 298, and 0.025–0.03 kg/s were recorded for the *φ*, solar intensity, temperature parameter, and mass flow rate, respectively. Energetic efficiency was enhanced as *φ*, mass flow rate, and solar irradiation increased and as temperature parameter reduced. However, after the peak energetic efficiency was attained, increasing *φ* and mass flow led to a decrease in value, while with solar intensity, the energetic efficiency remained constant. Entropy generation and pumping power ratio were found to increase with *φ*. In comparison with DIW, the energetic and exergetic efficiency and entropy generation drop of 23.47%, 9.26%, 12.65%, 18.05%, and 20.52%; 29.8%, 12.3%, 17.1%, 23.4%, and 25.1%; and 65.52%, 45.57%, 48.16%, 56.86%, and 57.44% were obtained for the DIW-based MWCNT, MgO, CuO, CuO-MWCNT, and MgO-MWCNT NFs, respectively. With the MWCNT/DIW NFs having the highest κ and lowest viscosity followed by MgO-MWCNT NFs and then CuO-MWCNT NFs, the best working fluids followed a similar trend (MWCNT > MgO-MWCNT > CuO-MWCNT).

Farajzadeh et al. [123] explored the thermal efficiency of utilizing DIW-based Al_2_O_3_-TiO_2_ (1:1) NFs (with *φ* = 0.1 wt% and 0.2 wt%) in an FPSC under varying volume flow rates (1.5–2.5 L/min). The thermal efficiency of all the samples was noticed to reduce as the temperature parameter increased while the temperatures at the inlet, outlet, and tank increased as solar radiation increased. Maximum thermal efficiency was recorded with Al_2_O_3_-TiO_2_/DIW NF at a flow rate of 2 L/m and 0.2 wt% concentration. At 0.1 wt% and in comparison, with DIW, efficiencies of 19%, 21%, and 26% were obtained for TiO_2_/, Al_2_O_3_/, and Al_2_O_3_-TiO_2_/DIW NFs, respectively. Increasing the concentration of the HNF (from 0.1 wt% to 0.2 wt%) led to a 5% enhancement of the thermal efficiency of the collector. Additionally, at flow rates of 2.0 L/m and 2.5 L/m, the thermal efficiency was noticed to be 8% and 5% above that of the flow rate of 1.5 mL, respectively. The heat loss parameter of DIW was the highest whereas that of HNFs was the lowest. The authors stressed that using HNFs of Al_2_O_3_-TiO_2_/DIW reduced the cost coupled with the higher thermal efficiency of the collector.

Okonkwo et al. [112] experimented the deployment of water-based Al_2_O_3_ and Al_2_O_3_-Fe_2_O_3_ NFs as working fluids in an FPSC. The thermodynamic performance (first and second laws) and optimization (of mass flow rate, *φ* (0.05–0.2 vol%), and temperature) were carried out. Their results showed that the exergy and energy efficiency, h, f, exergy destruction, absorbed energy parameter, exergy loss, and generation strongly depended on *φ*, mass flow rate, and temperature parameter. Energy efficiency was observed to increase as mass flow increased and as *φ* and temperature parameter decreased whereas the reverse was the case with exergy efficiency. Using 0.1 vol%, the HNF enhanced exergetic efficiency by 6.9% against 5.7% for the MNF while the energy efficiency was augmented by 2.16% for the MNF and depreciated by 1.79 for the HNF, as compared with water. Of the useful exergy (1123 W) absorbed from the sun using the collector, 73% was used up in the collector with the destruction of 59% of the total exergy. The h was observed to enhance as the temperature and mass flow rate increased with the MNF (72%) recording the highest value followed by the HNF (56%) and water. The reverse was noticed for f as the HNF was slightly higher than the MNF. This finding was due to the higher nano-size, viscosity, and density of HNF in comparison with the MNF despite the higher κ value.

Under varying mass fluxes (420 kg/s m^2^ and 598 kg/s m^2^) and mixing volume concentrations, the efficiency of MWCNT (0.003 vol% and 0.005 vol%) + Fe_3_O_4_ (0.01 vol% and 0.015 vol%) NFs as working fluids was investigated in an FPSC [117]. They noticed that the collector efficiency was improved as the mass flux and MWCNT concentration increased and Fe_3_O_4_ concentration decreased. The efficiency of water was 62.7% while those of the HNFs ranged from 73.5% to 80.3% and this translated to 17.2–28.1% above that of water. The use of MWCNT (0.005 vol%) + Fe_3_O_4_ (0.01 vol%) NF was observed to produce maximum efficiency. Similarly, increasing the mass flux of water enhanced the efficiency of the collector by 6.5% whereas that of MWCNT (0.005 vol%) + Fe_3_O_4_ (0.01 vol%) NF was improved from 74.5% to 80.3%.

Hussein et al. [118] examined the thermal efficiency of DW-based MWCNT-GNP-hBN (40:60) NFs (with *φ* = 0.05–0.1 wt%) as working fluids in FPSCs under varying flow rates (2–4 L/min). The result demonstrated that increasing the flow rate enhanced collector efficiency. Using 0.1 wt% HNF and at a flow rate of 4 L/min, the maximum collector efficiency of 85% was attained, which was 20% above that of DW. The highest enhancement for thermal heat gain and loss parameter was 21.9% and 78.3%, respectively, for 0.1 wt% MWCNT-GNP-hBN NF and at a flow rate of 4 L/min. A better performance was obtained for the solar collector using HNFs compared with MNFs and DW.

### 3.3. Parabolic Solar Collectors

Bellos and Tzivanidis [114] investigated the energetic and exergetic performance of Syltherm 800-based Al_2_O_3_ (3 vol%), TiO_2_ (3 vol%), and 1.5 vol% Al_2_O_3_ + 1.5 vol% TiO_2_ NFs deployed as thermal fluids in a parabolic solar collector under varying inlet temperatures (300–650 K). They observed that the thermal efficiency augmented with a decrease in inlet temperature while the exergetic efficiency, Nu, and h enhanced as the inlet temperature increased. The average thermal efficiency enhancement, Nu, and h of Al_2_O_3_ (3 vol%), TiO_2_ (3 vol%), and 1.5 vol% Al_2_O_3_ + 1.5 vol% TiO_2_ NFs were 0.340%, 0.341%, and 0.790% (0.33–1.80%); 23.5%, 23.8%, and 121.7%; and 34.9%, 35.2%, and 142.1%, respectively, compared with Syltherm 800. This translated to average h and Nu ratios of 2.4 and 2.2 and 1.35 and 1.23 for HNFs and MNFs, respectively. Using the HNF, exergetic efficiency of 38.35% was achieved against 37.94% for MNFs and 37.68% for Syltherm 800.

### 3.4. Vacuum Tube Solar Collectors

Lee et al. [117] experimented the deployment of MWCNT (0.003 vol% and 0.005 vol%) + Fe_3_O_4_ (0.01 vol% and 0.015 vol%) NFs as working fluids in a vacuum tube solar collector under varying mass fluxes (420 kg/s m^2^ and 598 kg/s m^2^) and mixing volume concentrations. The results demonstrated that the highest efficiency was achieved using MWCNT (0.005 vol%) + Fe_3_O_4_ (0.01 vol%) NF and at a mass flux of 598 598 kg/s m^2^. The HNFs recorded an efficiency range of 73.6–79.3% compared with 54.9% for water. They noticed that using both water and HNFs as working fluids, the FPSC recorded a higher efficiency compared to the vacuum tube solar collector under the same working condition. The heat gain revealed a 17.1–28.1% and 17.2–27.3% increase while the loss parameter recorded a 25.7–47.6% reduction and 6.93–17.1% improvement for the FPSC and vacuum tube collector, respectively. This result was mainly due to the characteristics of the working fluids, collector types, and operating conditions. Increasing the mass flux enhanced the efficiency of MWCNT (0.005 vol%) + Fe_3_O_4_ (0.01 vol%) NF from 73.7% to 79.8% and also increased the effectiveness of the vacuum tube collector. Under wide operating conditions, the vacuum tube collector was observed to perform better than the FPSC.

Salman et al. [121] investigated the thermal performance of DW-based Al-Al_2_O_3_ NFs having volume fractions of 1%, 3%, and 5% under increasing volume flow rates (15, 30, and 45 L/h) in a vacuum tube solar collector. They reported an improvement in thermal efficiency as volume fraction and flow rate increased. The maximum thermal efficiency of >60% was achieved at a flow rate of 45 L/h and a volume fraction of 5%, which was 24.89% higher compared with DW. Increasing the concentration of the HNF was also noticed to improve heat gain. At a flow rate of 15 L/h, the heat gain for water ranged from 40.46 W to 68.35 W, while at a flow rate of 30 L/h and volume fraction of 5%, a heat gain of 75.14–83.69 W was recorded for the HNF. The performance index of the HNF was found to be better than that of DW. Thus, deploying HNFs as working fluids in the vacuum tube collectors yielded improved thermal efficiency.

Under varying flow rates (0.56–1.35 L/min), Sundar et al. [85] examined the h, f, and collector thermal efficiency of DW-based ND-Co_3_O_4_ (67:33) NFs (*φ* = 0.05–0.15 wt%) as nano-coolants in an FPSC. The μ and κ of the hybrid nano-coolants were observed to enhance with *φ* and temperature increase. They showed that the Nu and h of the hybrid nano-coolants were improved with an increase in the flow rates, Re, and *φ* whereas the f was reduced as the *φ* and flow rate increased. Increasing the temperature parameter diminished the collector efficiency as increasing *φ* enhanced it. At a flow rate of 1.35 L/min, maximum Nu, h, f, and collector efficiency of 21.23%, 36.41%, 1.13-fold, and 59.78% were achieved using 0.15 wt% ND-Co_3_O_4_ nano-coolant, respectively, with the collector of efficiency of 49.81% recorded for DW. These peak values were obtained at 13:00 h in the day after which a decline was generally observed in these values. Additionally, correlations were proposed to estimate the Nu and f of the hybrid nano-coolants engaged in the FPSC.

### 3.5. Photovoltaic-Thermal Solar Collectors

The influence of mixing ratio (0.2–0.8), *φ* (0.01% and 1%), mass flow rate (0.01 kg/s and 0.1 kg/s), and solar irradiance on the energy (electrical and thermal) and exergy performance of water-Al_2_O_3_-ZnO NFs as thermal fluids in a PV/T solar collector was studied by Wole-Osho et al. [119]. The results proved that at an optimum mixing ratio of 0.47 of Al_2_O_3_ NPs in the HNF, exergy, thermal, and electrical efficiency of 15.13%, 55.9%, and 13.8%, respectively, was obtained for the PV/T collector. They noticed that the cell temperature of the PV/T diminished exponentially as the mass flow rate increased leading to a cell temperature reduction of 21% as the mass flow rate increased from 0.01 kg/s to 0.1 kg/s. At the maximum solar irradiation, cell temperatures of 37.5 °C and 46.8 °C were obtained for a mass fraction of 0.01% and flow rate of 0.01 kg/s while 37.8 °C and 47.8 °C were recorded for a 1% mass fraction and flow rate of 0.1 kg/s. The overall maximum thermal efficiency attained using water-Al_2_O_3_-ZnO NF in the PV/T collector was 91%, which translated to a 34% improvement over the use of water.

## 4. Thermal Energy Storage Application of Hybrid Nanofluids

**Figure 14:**
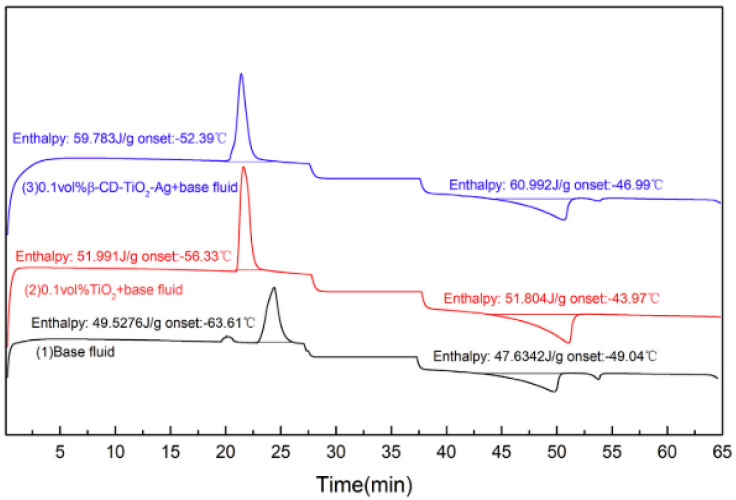
The DSC freezing–melting curves of 0.1 vil% β-CD-TiO_2_-Ag nanofluid, TiO_2_ nanofluid, and cooling medium (Adapted from Li et al. [83]).

Due to the ability of PCM to collect, store, and transfer latent thermal energy storage at high energy storage density under isothermal conditions, it is preferred to sensible thermal energy storage. However, the PCMs are known to possess very low κ, making them disadvantageous in terms of the reduction in their rate of energy stored and released. The advent of nanotechnology informed the addition of HNPs (with higher κ) to PCMs to enhance the κ of PCMs and thus, improve the efficiency and energy storage and release characteristics [111,124]. By incorporating heat storage into a solar collector, the overall efficiency is improved while minimizing the levelized energy cost of the system. Table 4 presents the summary of the thermal properties and performances of HNP-based thermal energy storage materials. The various HNPs (with different mixing ratios and nano-sizes) and base fluids engaged to formulate the different HNFs investigated for their thermal properties and performances as thermal energy storage materials were also provided in the table. In addition, the stability tests, process methods, surfactants, and the studied solar collectors are provided in the table. Noteworthy papers available in the open literature on the subject from the inception of the study to date were used to compile Table 4. The melting and freezing curves of an HNF (β-CD-TiO_2_-Ag) are presented in Figure 14.

Chieruzzi et al. [125] experimentally explored the thermal storage performance of binary salt (NaNO_3_-KNO_3_)-based SiO_2_-Al_2_O_3_ (82:18) NFs with *φ* = 0.5–1.5 wt% prepared using the direct method. They observed that the 1 wt% SiO_2_-Al_2_O_3_ NF yielded the highest heat of fusion (127.2 kJ/kg) and lowest solidification (209.3 °C) and melting (223.9 °C) temperature compared with Al_2_O_3_, SiO_2_, and TiO_2_ NFs. Additionally, the highest stored energy was noticed with 1 wt% SiO_2_-Al_2_O_3_ NF. The doping of the binary salt with SiO_2_-Al_2_O_3_ NPs using *φ* = 1 wt% enhanced the specific heat by 57% (solid phase) and 22% (liquid phase), and reduced the melting temperature by 8 °C and solidification temperature by 10 °C. The improvement of the specific heat capacity of SiO_2_-Al_2_O_3_/binary salt NF was noticed to encourage its utilization as an energy storage medium in concentrated solar power plants, which could lessen the quantity of the storage media and the cost of electricity.

Harikrishnan et al. [78] examined the thermal properties and energy storage performance of paraffin-based CuO-TiO_2_ (50:50) NFs with *φ* = 0.25–1 wt%. They observed that the κ and μ of CuO NFs were highest, followed by CuO-TiO_2_ NFs and then TiO_2_ NFs. Increasing *φ* increased κ, μ, FT, and MT, and reduced MLH and FLH. The FT and MT of the HNFs ranged from 56.54 °C to 56.96 °C and 60.34 °C to 60.84 °C compared with 56.47 °C and 60.23 °C for paraffin, respectively. Additionally, the FLH and MLH of 1 wt% CuO-TiO_2_ NF were 182.7 kJ/kg and 190.1 kJ/kg compared with 189.5 kJ/kg and 197.6 kJ/kg for paraffin, which corresponded to 1.83% and 2.27% reduction, respectively. After 2000 cycles to examine the thermal stability of the HNF-based energy storage materials, the melting and freezing processes were achieved between 8–11 min and 16–22 min, respectively, with 1 wt% CuO-TiO_2_ NF recording the lowest time. A reduction of 29.8% (melting time) and 28.7% (freezing time) was attained using 1 wt% CuO-TiO_2_ NF.

Organic ester-based functionalized Ag-TiO_2_ NFs with *φ* = 0.1–1.5 wt% were examined by Parameshwaran et al. [81] for their thermal properties and thermal energy storage characteristics. Increasing *φ* caused a direct improvement of μ (0.35–3.8%) and κ (10–52%) while it diminished latent heat capacity and enthalpy of latent heat. The HNFs were observed to be thermally more stable than the organic ester, with maximum mass loss at 191 °C for 0.8 wt% Ag-TiO_2_ HNF and 179 °C for the organic ester. After 1000 melting and freezing cycles, the 0.8 wt% HNF possessed a latent heat capacity of 90.69 kJ/kg (1.00–9.18% reduction) and 87.69 kJ/kg (1.74–7.38% reduction) and onset temperature of 6.91 °C and 6.83 °C, compared with 95.60 kJ/kg and 90.70 kJ/kg and 6.80 °C and 6.75 °C obtained for organic ester during melting and freezing processes, respectively. The supercooling degree of 0.8 wt% HNF was 1.82 °C while that of the organic ester was 2.07 °C. It was noticed that in comparison with the pure organic ester, the duration of the onset of melting and freezing for the Ag-TiO_2_ NFs declined by 1.7–8.5% and 5.1–23.9%, respectively. Owing to the enhanced thermal properties and thermal heat storage performance of the ester-based Ag-TiO_2_ NFs, the potential application in buildings for thermal storage cooling was proposed.

The dual feasibility of Sn-SiO_2_/Ag NPs as a working fluid and energy storage material for DASC was investigated by Zeng et al. [126]. The Sn-SiO_2_/Ag NPs were observed to produce an enhanced optical absorption in comparison with Sn-SiO_2_ NPs. They reported temperature and enthalpy values of 230.5 °C and 57.7 J/g, 230.3 °C and 47.5 J/g, and 227.1 and 36.0 J/g for the melting of Sn, Sn-SiO_2_, and Sn-SiO_2_/Ag NPs, respectively. Similarly, temperature and enthalpy values of 123.9 °C and 49.1 J/g, 126.0 °C and 39.5J/g, and 128.0 °C and 29.5J/g were obtained for the freezing of Sn, Sn-SiO_2_, and Sn-SiO_2_/Ag NPs, respectively. These results showed that the phase change temperatures of Sn-SiO_2_ and Sn-SiO_2_/Ag NPs were very close to that of Sn NPs due to insignificant changes in the thermal property. The thermal storage efficiency of the encapsulated Sn in Sn-SiO_2_ and Sn-SiO_2_/Ag NPs was 99.0% and 98.4%, respectively. Thus, indicating that the encapsulated Sn efficiently stored and released the latent heat via phase changes. Under 200 heating–cooling cycles to estimate the thermal stability of Sn-SiO_2_/Ag NPs, 227.0 °C and 127.8 °C and 35.7 J/g and 29.2 J/g were recorded as the melting and freezing temperatures and enthalpies, respectively. In addition, the enhancement of volumetric thermal energy storage was found to reduce with a range of operating temperatures and increased with volume fraction. The utilization of Sn-SiO_2_/Ag NPs in DASC was reported to effectively enhance thermal and energy storage performance under medium-high operating temperatures.

As a follow-up study to the work of Chieruzzi et al. [125], Chieruzzi et al. [127] prepared binary salt (NaNO_3_-KNO_3_)-based SiO_2_-Al_2_O_3_ (82:18) NF with *φ* = 1 wt% using a micro-compounder (twin-screw) under varying stirring rates (100 and 200 rpm) and stirring durations (15 and 30 min) at a high temperature (300 °C) and investigated the thermal energy storage performance. Their results demonstrated that at the higher stirring rate (200 rpm) and duration (30 min), maximum c_p_ (2.42 J/g °C–solid phase and 1.94 J/g °C–liquid phase) and stored energy (373 J/g) were obtained. The specific heat of the SiO_2_-Al_2_O_3_/binary salt NF was improved by 52.1% (solid phase) and 18.6% (liquid phase), the heat of fusion was enhanced by 1.5–7.4%, and the stored energy was augmented by 13.5% while the solidification temperature was reduced up to 9.7 °C, compared with the binary salt. The study showed that engaging the direct method improved the energy storage characteristics of 0.1 wt% SiO_2_-Al_2_O_3_/binary salt NF over the high-temperature mixing technique.

**Table 4:** Summary of stability tests, surfactants, process methods, thermal properties, and performances of different HNFs deployed in various solar collectors as thermal energy storage.

References	HNF (Mix)/Base Fluid	*φ*	Application	Nano-Size (nm)	Stability	Thermal Properties	Result
Liu et al. [111]	GO-CNT (3:1, 1:1, and 3:1)/ MEPCM/DIW	0.1–0.6 wt%		GO-CNT-50	ZP, UV, SDS, (2-step)	Κ	The latent heat of GO-CNT (3:1, 1:1, and 1:3)/MEPCM-DIW NFs was slightly reduced compared with MEPCM alone. The HNF with a mixing ratio of 3:1 yielded the highest κ enhancement (195%).
Harikrishnan et al. [124]	Ni-ZnO/oleic acid	0.3–1.2 wt%		Ni-ZnO-36	SDBS (2-step)	Κ	The time taken by melting (900 s–1280s) and solidification (990 s–1385s) processes was lower for oleic acid-Ni-ZnO NFs than oleic acid. Oleic acid-Ni-ZnO NFs recorded κ enhancement of 25.43–87.27% relative to oleic acid.
Shao et al. [128]	TiO_2_-NT and TiO_2_-NPT (0:100–100:0)/DIW	0.1–0.3 wt%		TiO_2_-32 NT-10 and NPT-50–80	(2-step)	θc and κ	The enhancement κ by 54.91% and 56.42% for DIW-based TiNTs-TiNPTs NFs was responsible for the reduction of supercooling temperature and solidification time by 4.97 °C and 5.27 °C, and 54.91% and 56.42%, respectively, as compared with TiNT and TiNPT NFs.
Abdullah et al. [129]	CuTsPc-TiO_2_/water	-		-	-	-	The capacitive and resistive sensitivity of TiO_2_-CuTsPc NF was 5.548 nF/°C and 0.098 kΩ/°C while that of CuTsPc was 1.064 nF/°C and 0.23 kΩ/°C. This revealed the capacitance switching of the device.
Zeng et al. [126]	Sn-SiO_2_/Ag	-	DASC	SiO_2_-10 Sn-68	UV, PVP (2-step)	-	The thermal storage efficiency of Sn in Sn/SiO_2_ and Sn/SiO_2_/Ag NPs was 99.0% and 98.4%, respectively. Under 200 heating–cooling cycles, 227.0 °C and 127.8 °C and 35.7 J/g and 29.2 J/g were recorded as the melting and freezing temperatures and enthalpies, respectively.
Chieruzzi et al. [125]	SiO_2_-Al_2_O_3_ (82:18)/NaNO_3_- KNO_3_ (60:40)	0.5–1.5 wt%	CSP	SiO_2_-Al_2_O_3_- 2–200 SiO_2_- 7 Al_2_O_3_- 13	(2-step)	c_p_, heat of fusion, MT, ST, storage energy	0.1 wt% binary salt-based SiO_2_-Al_2_O_3_ NF improved the specific heat by 57% (solid phase) and 22% (liquid phase), and reduced melting temperature by 8 °C and solidification temperature by 10 °C. HNF was better than SiO_2_, Al_2_O_3_, and TiO_2_ NFs.
Chieruzzi et al. [127]	SiO_2_-Al_2_O_3_ (82:18)/NaNO_3_- KNO_3_ (60:40)	1 wt%	CSP	SiO_2_-Al_2_O_3_-2–200 SiO_2_- 7 Al_2_O_3_- 13	(2-step)	c_p_, heat of fusion, MT, ST, storage energy	The c_p_ of SiO_2_-Al_2_O_3_/binary salt NF was improved by 52.1% (solid phase) and 18.6% (liquid phase), the heat of fusion was enhanced by 1.5–7.4%, and the stored energy was augmented by 13.5% in comparison with the binary salt.
Harikrishnan et al. [78]	CuO-TiO_2_ (50:50)/paraffin	0.25–1 wt%	-	CuO-TiO_2_- 21	SDBS, Visual	κ, μ, MT, FT, MLH, FLH	FLH and MLH of 1 wt% CuO-TiO_2_ NF were reduced by 1.83% and 2.27%, respectively, compared with the base fluid. A reduction of 29.8% (melting time) and 28.7% (freezing time) was achieved using 1 wt% CuO-TiO_2_ NF.
Vaka et al. [130]	GO-TiO_2_/hybrid eutectic salt	0.01–0.1 wt%	CSP		-	-	The c_p_ of GO-TiO_2_/eutectic material was improved by 9.8%, 19.1%, and 19.6% for 0.01, 0.05, and 0.1 wt%, compared with the hybrid eutectic salt. The highest c_p_, heat flow, and latent heat were attained with 0.05 wt% GO-TiO_2_/eutectic material.
Parameshwaran et al. [81]	Ag-TiO_2_/organic ester	0.1–1.5 wt%	Buildings internal walls	Ag-TiO_2_- 10–95	Ethanol (2-step)	-	The duration of the onset of melting and freezing for the Ag-TiO_2_ NFs declined by 1.7–8.5% and 5.1–23.9%, respectively, compared with pure organic ester. After 1000 cycles, the latent heat capacity of the 0.8 wt% HNF was reduced by 1.00–9.18% (melting) and 1.74–7.38% (freezing).
Parameshwaran et al. [82]	Cu-TiO_2_/pristine	0.02–0.1 wt%	Buildings internal walls	-	PVP and ethanol (2-step)	-	Adding Cu-TiO_2_ nanomaterial into pristine enhanced κ up to 0.08 wt% (0.1926 W/m K). The average enthalpy of latent heat of pristine-based Cu-TiO_2_ nanomaterials was 190.03 J/g (freezing) and 195.03 J/g (melting), similar to that of pristine.
Li et al. [83]	β-CD-TiO_2_-Ag/EG-DIW (40:60)	0.025–0.1 vol%	Cold energy storage systems	β-CD-TiO_2_-Ag–40–50 TiO_2_-Ag–40–50 TiO_2_ -25–30	ZP (2-step)	-	The 0.1 vol% β-CD-TiO_2_-Ag PCM yielded higher melting phase change temperature, supercooling temperature, freezing phase change temperature, freezing phase enthalpy, and melting phase enthalpy and lower supercooling degree and total freezing time than the pure PCM.
Nithiyanantham et al. [84]	SiO_2_-Al_2_O_3_/binary nitrate salt (eutectic)	1 wt%	CSP	SiO_2_-Al_2_O_3_–12, 14, 17	-	μ, κ, thermal diffusivity	At temperatures of 250 °C–400 °C, the thermal diffusivity of 10-SiO_2_-Al_2_O_3_ nano-PCM, 20-SiO_2_-Al_2_O_3_ nano-PCM, and 35-SiO_2_-Al_2_O_3_ nano-PCM were improved by −8%–−4%, 0%–−2%, and 7–14%, respectively, compared with eutectic-based PCM.
Sharma et al. [131]	CoZnFe_2_O_4_/paraffin	0.1 wt%	-	CoZnFe_2_O_4_- 30-40	-	-	The discharging of the paraffin wax took 100 min (33 °C) with CoZnFe_2_O_4_ NF and 130 min (35 °C) engaging DW. The charging and discharging time declined by 25% and 23%, respectively, for the paraffin wax using CoZnFe_2_O_4_ NF.

The thermal property and behavior of DIW-based TiNTs-TiNPTs (0:100–100:0) NFs with *φ* = 0.1–0.3 wt% as cold thermal energy storage materials were investigated by Shao et al. [128]. Authors observed that for both HNFs and MNFs, the supercooling temperature and solidification time surged as solidification–melt cycles increased. Owing to the larger surface area and high κ, the HNFs exhibited lower supercooling temperatures and lesser solidification time compared with MNFs. The enhancement of κ by 54.91% and 56.42% for DIW-based TiNTs-TiNPTs NFs was observed to be responsible for the reduction of supercooling temperature and solidification time by 4.97 °C and 5.27 °C, and 54.91% and 56.42%, respectively, when compared with TiNT and TiNPT NFs. The latent heat of DIW-based TiNTs-TiNPTs NFs (*φ* = 0.1–0.3 wt%) was lowered by 9.21% (303.4 kJ/kg–269.2 kJ/kg) and 4.72% (298.7 kJ/kg–284.6 kJ/kg) for TiNTs-TiNPTs (75:25) and TiNTs-TiNPTs (50:50) NFs, respectively. In addition, the latent heat of TiNTs-TiNPTs (75:25) and TiNTs-TiNPTs (50:50) NFs for *φ* = 0.1 wt% was reduced by 10.93% and 9.43%, respectively, relative to DIW.

Liu et al. [111] experimented the utilization of MEPCM-DIW-based GO, CNT, and GO-CNT (mixing ratios of 1:1, 1:3, and 3:1) NF fillers with *φ* = 0.1–0.6 wt% for solar energy application. These materials were investigated for their κ, phase change properties (thermal reliability and stability), absorption and optical properties, and PTEC characteristics. They reported that MEPCM-DIW-based GO-CNT (3:1) NF with *φ* = 0.6 wt% has the highest κ enhancement (195%) compared with GO/MEPCM-DIW NFs, CNT/MEPCM-DIW NFs, and MEPCM. Additionally, good optical and absorption properties and PTEC performance were observed using MEPCM-DIW-based GO-CNT NFs. The phase change properties revealed that the latent heat of GO-CNT (3:1, 1:1, and 1:3)/MEPCM-DIW NFs was slightly reduced compared with MEPCM alone.

For concentrated solar power applications, the thermal energy storage characteristics of binary eutectic-based SiO_2_-Al_2_O_3_ nano-PCM with SiO_2_ outer layer thickness of 10, 20, and 35 were investigated by Nithiyanantham et al. [84]. They observed that at a temperature range of 250–400 °C, the thermal diffusivity, κ, and μ of 10-SiO_2_-Al_2_O_3_ nano-PCM, 20-SiO_2_-Al_2_O_3_ nano-PCM, and 35-SiO_2_-Al_2_O_3_ nano-PCM were enhanced by −8–−4%, 0–−2%, and 7–14%; −6–−2%, 3–−1%, and 11–19%; and 16–25%, 16–30%, and 25–34%, respectively when compared with eutectic-based PCM. All the tested samples were noticed to be thermally stable before 565 °C with equal decomposition temperature. Thus, indicating no influence of the operating temperature of the eutectic-based PCM on the SiO_2_-Al_2_O_3_ nano-PCMs. These results portend the ability of SiO_2_-Al_2_O_3_ nano-PCMs to improve heat transfer efficiency and reduce the levelized cost of electricity related to concentrated solar power applications.

Harikrishnan et al. [124] examined the deployment of oleic acid-Ni-ZnO NFs with *φ* = 0.3–1.2 wt% as cool thermal energy storage materials. They revealed that increasing *φ* caused the enhancement of κ and phase change temperature, and a reduction of latent heat. At *φ* = 1.2 wt%, peak phase change temperature (improvement) and latent heat (reduction) of −1.13% and 1.34%, and 1.91% and 2.23% for melting and solidification processes were recorded, respectively. These results can be linked to the structure, size, and *φ* of the Ni and CuO NPs and the existence of strong chemical interaction within oleic acid-Ni-ZnO NFs. An increase in the thermal cycle was noticed to augment phase change temperature and decrease latent heat. Under varying thermal cycles and at *φ* = 1.2 wt%, the highest difference in phase change temperature and latent heat was 1.62% and −1.54% and 1.51% and 1.62% for solidification and melting processes, respectively. These values indicated that oleic acid-Ni-ZnO NFs possessed better thermal reliability than pure oleic as PCM for long-term operation. The time taken by melting (900–1280 s) and solidification (990–1385 s) processes was observed to be lower for oleic acid-Ni-ZnO NFs than oleic acid. Oleic acid-Ni-ZnO NFs were recorded to have κ improved by 25.43–87.27% relative to oleic acid.

Thermal energy storage characteristics (latent heat, c_p_, heat flow, and thermal stability) of hybrid eutectic salt-based GO-TiO_2_ material with *φ* = 0.01–0.1 wt% was investigated by Vaka et al. [130]. They demonstrated that up to 580 °C, the GO-TiO_2_/eutectic material was observed to be thermally reliable. Maximum c_p_, heat flow, and latent heat were achieved with 0.05 wt% GO-TiO_2_/eutectic material. In comparison with the hybrid eutectic salt (1.342 J/g), the c_p_ of GO-TiO_2_/eutectic material was enhanced by 9.8%, 19.1%, and 19.6% for concentrations of 0.01, 0.05, and 0.1 wt%. In addition, the latent heat and c_p_ of 83.74 J/g and 1.342 J/g, 41.74 J/g and 1.47 J/g, 52.25 J/g and 1.606 J/g, and 35.21 J/g and 1.599 J/g were obtained for hybrid eutectic salt, 0.01, 0.05, and 0.1 wt% GO-TiO_2_/eutectic material, respectively.

Parameshwaran et al. [82] experimented the development of mortar-embedded thermal energy storage materials formulated using pristine-based Cu-TiO_2_ nanomaterials with *φ* = 0.02–0.1 wt% for passive cooling of buildings. They showed that pristine-based Cu-TiO_2_ nanomaterials were observed to be thermally more stable than the pristine at a higher temperature (>100.4 °C). The doping of Cu-TiO_2_ nanomaterial into pristine was noticed to enhance κ up to 0.08 wt% (0.1926 W/m K), leading to an enhancement of 5.53%. The average enthalpy of latent heat for the pristine-based Cu-TiO_2_ nanomaterials was 190.03 J/g (freezing process) and 195.03 J/g (melting process) and was found to be consistent with that of pristine. Onset temperatures of 21 °C (freezing process) and 22 °C (melting process) were obtained for both the pristine-based Cu-TiO_2_ nanomaterials and pristine, respectively. On adding mortar to pristine-based Cu-TiO_2_ nanomaterials, the resultant materials showed a decline in the compressive strength ranging from 21.7 MPa to 20.2 MPa, compared with pure mortar (22.3 MPa). This finding revealed the potential utilization of pristine-based Cu-TiO_2_ nanomaterials as an additive to mortar for indoor wall plastering to serve as a passive thermal energy storage material in building cooling without compromising structural stability.

An eco-friendly and novel hybrid nano-PCM for cold thermal energy systems application was studied by Li et al. [83]. They examined the thermal properties and thermal storage behaviors of functionalized β-CD-TiO_2_-Ag/EG-DIW (40:60) PCM with *φ* = 0.025–0.1 vol%. Results proved that the 0.1 vol% β-CD-TiO_2_-Ag PCM possessed a higher melting phase change temperature (−46.99 °C), supercooling temperature (−52.39 °C), freezing phase change temperature (−52.39 °C), freezing phase enthalpy (59.78 J/g), κ (42.17% improvement), melting phase enthalpy (60.99 J/g), lower supercooling degree (5.4 °C), and total freezing time (179.4 s) than the pure PCM. These implied that the 0.1 vol% hybrid nano-PCM was thermally more stable, has higher κ, improved enthalpy, lower supercooling degree, and freezing time compared with pure PCM. The functionalization of TiO_2_ was noticed to impact positively on the thermal properties and characteristics of the β-CD-TiO_2_-Ag PCM. The deployment of 0.1 vol% β-CD-TiO_2_-Ag PCM for cold thermal energy storage applications was envisioned to reduce the environmental pollution footprint and increase the efficiency of energy storage.

Sharma et al. [71] studied the latent heat storage characteristics of paraffin wax (as PCM) through charging with DW-CoZnFe_2_O_4_ (*φ* = 0.1 wt%) NF and DW as thermal fluids. The results demonstrated that at a heating temperature of 90 °C and initial paraffin wax of 33 °C, the paraffin wax was heated to 60.32 °C and 62.01 °C for 120 min and 90 min using DW and CoZnFe_2_O_4_ NF, respectively. The discharging of the paraffin wax took 100 min (33 °C) using CoZnFe_2_O_4_ NF and 130 min (35 °C) engaging DW. The charging and discharging time reductions of 25% and 23%, respectively, were obtained for the paraffin wax using CoZnFe_2_O_4_ NF. This implied that CoZnFe_2_O_4_ NF was better used for charging while DW was preferred for discharging concerning improving the efficiency of thermal energy storage systems.

## 5. Challenge and Research Outlook

**Figure 15:**
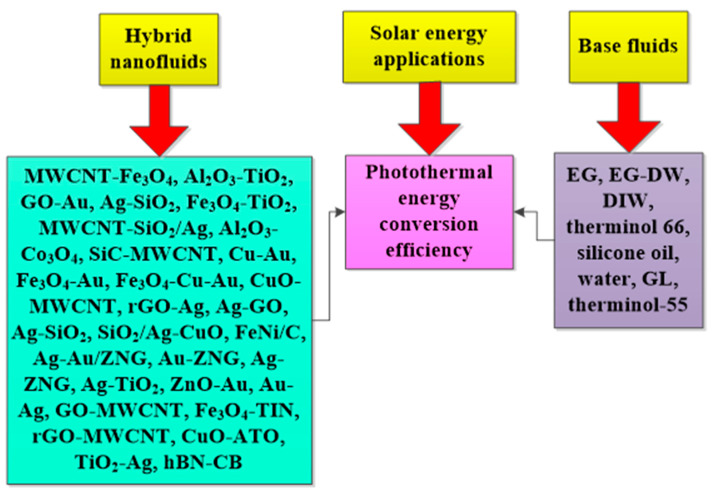
Hybrid nanofluids deployed for photothermal energy conversion efficiency studies.

**Figure 16:**
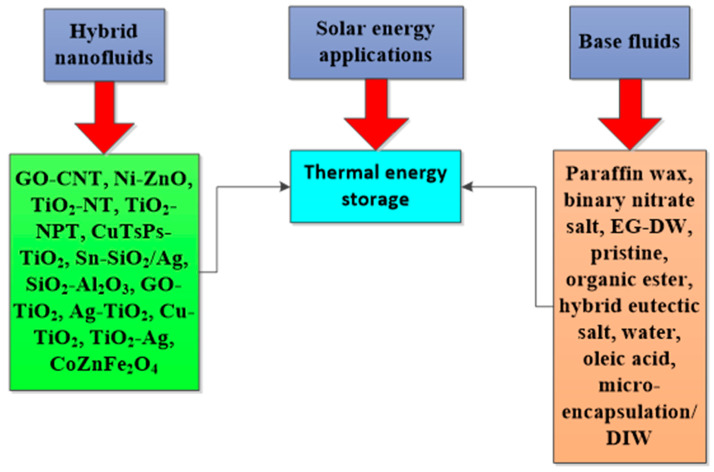
Hybrid nano-materials deployed as thermal energy storage materials.

**Figure 17:**
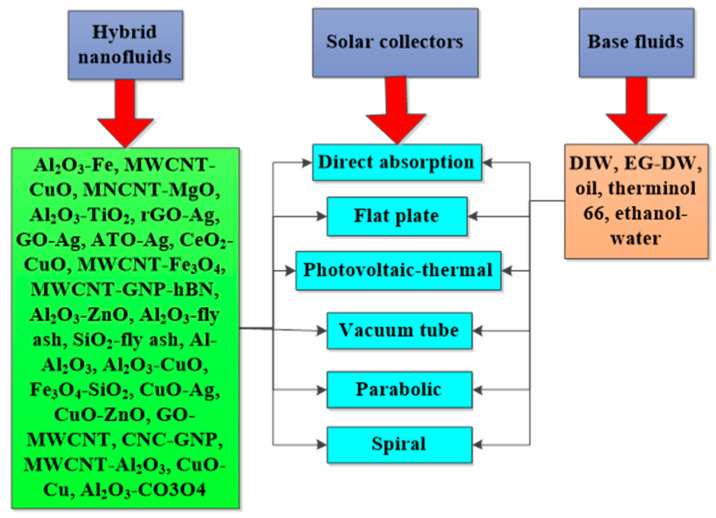
Hybrid nanofluids deployed in various solar collectors.

Subject to the reviewed publications on the experimental studies involved in the applications of HNFs as energy-efficient thermal media in solar energy collectors and thermal energy storage. Gaps observed for future research have been discussed herein. Figure 15, Figure 16 and Figure 17 provide the state of experimental studies conducted on the utilization of HNFs in solar energy and thermal energy storage applications.

Generally, it is observed that the suspension of different HNPs in various base fluids improved the optical and thermophysical properties, which consequently enhanced the relevant parameters such as SWEA, PTEC, thermal energy storage, and exergetic performances, depending on the application. These findings are found to be strongly connected to the volume/mass concentration, mixing ratio, stability, optical and thermophysical properties of HNPs and base fluids, formulation method, and magnetic field strength and orientation. It is worth emphasizing that the good stability of formulated HNFs considerably enhanced the stability, optical, and thermophysical properties and finally improved the performance of the relevant application [81,83]. This goes to confirm the serious need to obtain a moderately stable HNF prior to further experimental activities. Although some of the reviewed studies reported the stability of the formulated HNFs, some failed in this regard. For experimental studies involving HNFs, stability determination is very crucial as failure to carry out such is an indication that the results obtained thereafter are susceptible to inaccuracy [132,133,134]. The use of a non-scientific visual method (alone) to check stability is not adequate as some studies only engaged this technique.

A salient observation of the works of Yang et al. [135], Hou et al. [136], Li et al. [137], He et al. [138], Luo et al. [139], and Du et al. [140] revealed that the modification of HNPs, deployment of surfactants, and direct utilization of HNPs for HNFs formulation improved the stability, which further enhanced the performance in various applications. Du et al. [140] and Hou et al. [136] also showed that the dilution of MNFs to form HNFs yielded lower stability and under-performed compared to the formulation of HNFs from the use of HNPs. However, the reverse is reported by Hong et al. [115] for the HNF of rGO and Ag. This is considered a subject for further research in order not to undermine the performance of HNFs in different applications.

With 28 HNFs and eight base fluids (Figure 15), 13 HNF-based energy storage materials and nine base fluids (Figure 16), and 18 hybrid nano-coolants and three base fluids (Figure 17), deployed for PTEC, thermal energy storage, and solar energy, respectively, it reveals the need to intensify experimental studies in this respect. By comparing Figure 15 to Figure 17, many HNFs have been studied for their PTEC performance but limited works have been carried out on their applications as nano-coolants in various types of solar collectors. In general, and considering Figure 15, Figure 16 and Figure 17, it can be that more and different HNPs combinations and base fluids needed to be investigated in future studies. Presently, ionic base fluids are yet to be investigated concerning the HNFs application focus of this review. In addition, green base fluids and green HNFs under the auspices of green, efficient, and sustainable HNFs are futuristic experimental undertakings in this research direction as very few studies have been carried out on the thermophysical and convective properties [141,142,143,144,145,146,147,148].

Additionally, the exposure of HNFs, especially the magnetic types, to magnetic fields has been reported to enhance their thermal properties and convective characteristics [57,149,150,151,152,153,154]. In the context of this present study, only the works of Shin et al. [77], Shi et al. [59], Zeng and Xuan [94], and Wang et al. [99] have deployed magnetic fields (at varying intensities and orientations) to passively augment the PTEC performance of HNFs as working fluids for solar energy application. The possibility of controlling the exergetic and energetic performance of HNF-based coolants for solar energy applications especially is yet to be explored experimentally. The same goes for the magnetic field influence on the thermal energy storage characteristics of HNF-based thermal storage materials. With very scarce documentation on the low-temperature application of HNFs, future studies are recommended concerning solar refrigeration.

Subject to the utilization of HNPs and the respective base fluids as energy-efficient thermal media in solar energy and thermal energy storage applications to reduce cost and save energy by increasing efficiency, environmental and health issues remain a challenge. In agreement with the global outcry and pursuit of sustainability and greening in all its ramifications, eco-friendly bio-sourced HNPs and base fluids appear to be the future research focus.

## 6. Conclusions

Experimental studies on the applications of HNF as solar nano-coolants and thermal energy storage materials have been critically reviewed. Most of the studies revealed that the HNF yielded better performances than MNF and traditional fluids in the various applications and thus, saved energy as a result of the improved efficiency. Hybrid nano-coolants in different solar collectors mainly demonstrated reduced exergy efficiency and increased energy efficiency. For the HNF-based PCM, both the discharging and charging time are reduced compared with the PCM coupled with improved thermal energy storage performance. These results are strongly dependent on the types of HNPs and mixing ratios, types of base fluids, nano-size of HNPs, thermal and optical properties, flow regime, and *φ*, subject to respective applications. Further experimental studies in line with these applications coupled with the influence of magnetic and electric fields on their performances are expected in the nearest future. Green HNPs and base fluids are future biomaterials for HNF formulation in answer to sustainable and efficient thermal transport media.

## Figures and Tables

**Table 3:** Summary of stability tests, thermal properties, and performances of different HNFs, deployed in various solar collectors.

References	HNF (Mix)/Base Fluid	*φ*	Collector Type	Nano-Size (nm)	Stability	Thermal Properties	Result
Okonkwo et al. [112]	Al_2_O_3_-Fe (1:1)/W	0.05–0.2 vol%	Flat plate (*φ*, mass flow rate, and temperature parameter)	Al_2_O_3_-29 Fe-46 Al_2_O_3_-Fe-84	ZP	κ, μ, and c_p_	With 0.1 vol%, the exergetic efficiency of MNF and HNF was enhanced by 5.7% and 6.9% while the energy efficiency was augmented by 2.16% and depreciated by 1.79%, respectively, as compared with water. The h was observed to enhance as the mass flow rate and temperature increased, with MNF (72%) recording the highest value followed by HNF (56%) and water.
Verma et al. [113]	MWCNT-CuO and -MgO (20–80)/DIW	0.25–2 vol%	Flat plate (*φ*, mass flow rate, solar intensity, and temperature parameter)	CuO-42 MgO- MWCNT-7	UV	κ, μ, *ρ*, and c_p_	In comparison with DIW, the energetic and exergetic efficiency of 23.47%, 9.26%, 12.65%, 18.05%, and 20.52%, and 29.8%, 12.3%, 17.1%, 23.4%, and 25.1% were obtained for the DIW-based MWCNT, MgO, CuO, CuO-MWCNT, and MgO-MWCNT NFs, respectively.
Bellos and Tzivanidis [114]	Al_2_O_3_-TiO_2_ (1.5:1.5 vol%)/oil	3 vol%	Parabolic trough (inlet temperatures)	-	-	-	Thermal efficiency augmented with a decrease in inlet temperature while the exergetic efficiency, Nu, and h enhanced as the inlet temperature increased. Using the HNF, exergetic efficiency of 38.35% was achieved against 37.94% for MNFs and 37.68% for Syltherm 800.
Hong et al. [115]	rGO-Ag, rGO +Ag, GO-Ag, GO + Ag/water	-	DASCs	Ag-10–20	UV	-	An order of rGO + Ag NFs > GO + Ag NFs > rGO-Ag NFs > GO-Ag NFs was noticed for the water mass loss, evaporation rate, and relative efficiency. At 3 runs, the relative efficiency was 73.2–91.6% for the rGO + Ag NFs with 1, 0.45, 0.225, and 0.1125 mg/mL, respectively.
Sreekumar et al. [63]	ATO/Ag/DIW	0.1 wt% (optimized)	PTDASC	ATO-Ag-20–50 Ag-10	UV, SDS, Visual (2-step)	-	Using HNF at a mass flow rate of 0.022 kg/s led to a peak thermal efficiency of 63.5% and the highest exergy efficiency of 5.6%. Optical efficiency was improved by 75% at 0°.
Mohan and Sajeeb [116]	CeO_2_-CuO (1:0–0:1)/DIW	0.1 vol%	DASC	CeO_2_-30–50 CuO-30–50	Visual	-	At a flow rate of 100 cc/min, the thermal efficiency of 13.8%, 18.1%, 24.3%, 24.9%, and 26.1% was obtained for the HNFs with mixing ratios of 1:0, 1:0.5, 1:1, 0.5:1, and 0:1, respectively, compared with DIW.
Lee et al. [117]	MWCNT-Fe_3_O_4_/W	0.003 and 0.005 vol% (MWCNT), 0.01 and 0.05 vol% (Fe_3_O_4_)	Flat plate	MWCNT-20 Fe_3_O_4_-30	ZP, Visual	-	The efficiency of water was 62.7% while those of the HNFs were 73.5–80.3% and this translated to 17.2–28.1% above that of water. The use of MWCNT (0.005 vol%) + Fe_3_O_4_ (0.01 vol%) NF produced the highest efficiency.
Lee et al. [117]	MWCNT-Fe_3_O_4_/W	0.003 and 0.005 vol% (MWCNT), 0.01 and 0.05 vol% (Fe_3_O_4_)	Vacuum tube	MWCNT-20 Fe_3_O_4_-30	ZP, Visual	-	At a mass flux of 598 kg/s m^2^, maximum efficiency was attained using MWCNT (0.005 vol%) + Fe_3_O_4_ (0.01 vol%) NF. The HNFs recorded an efficiency of 73.6–79.3% compared with 54.9% for water.
Hussein et al. [118]	MWCNT-GNP-HBN (40:60)/DIW	0.05, 0.08, and 0.1 wt%.	Flat plate	MWCNT-15 GNP- 2 μm	UV, ZP, Tween (1:1)	μ, c_p_, and κ	At a flow rate of 4 L/min, the highest collector efficiency of 85% was achieved with 0.1 wt% HNF, which was 20% higher than DW.
Wole-Osho et al. [119]	Al_2_O_3_-ZnO (1:2, 1:1, and 2:1)/W	0.01% and 1%	PV-T	Al_2_O_3_-29 ZnO-70	-	μ, c_p_, and κ	Using an optimum mixing ratio of 0.47 of Al_2_O_3_ NPs in the HNF, the exergy, thermal, and electrical efficiency of the PV/T collector was 15.13%, 55.9%, and 13.8%, respectively. The overall maximum thermal efficiency of water-Al_2_O_3_-ZnO NF for the collector was 91%.
Thakur et al. [120]	Al_2_O_3_-fly ash and SiO_2_-fly ash (80:20)/DW	0.5–2 vol%	Microchannel-based DASC	Fly ash-88 Al_2_O_3_-30 SiO_2_-60	DLS, ZP, Sodium oleate (2-step)	μ, c_p_, *ρ*, and κ	The thermal and exergy efficiency of the collector was 72.82% and 59.23% and 73% and 68.09% for Al_2_O_3_-fly ash (80:20) and SiO_2_-fly ash (80:20) NFs, respectively. Pumping power of Al_2_O_3_-fly ash (80:20) and SiO_2_-fly ash (80:20) NFs was higher than DW.
Salman et al. [121]	Al-_Al2O3/_DW	1%, 3%, and 5%	Vacuum tube	Al-50 Al_2_O_3_-50	-	-	Peak thermal efficiency was >60% at a flow rate of 45 L/h and volume fraction of 5%, which was 24.89% higher compared with DW.
Tahat and Benim [122]	Al_2_O_3_-CuO (70:30)/EG-W (25:75 wt%	0.5–2 vol%	Flat plate solar collector	Al_2_O_3_-40 CuO-29	ZP (2-step)	μ, *ρ*, and κ	Thermal efficiency of FPSC was 42–52% as the volume fraction increased from 0.5–2% when compared with water.
Mendari et al. [75]	Al_2_O_3_-CuO/ EG-DW (50:50) and DW	CuO-0.001% and Al_2_O_3_-0.04%	DAPTSC	Al_2_O_3_-40 CuO-100	UV, Visual, pH, SHMP (2-step)	Absorbance and κ	Flow rate increase reduced temperature change and outlet temperature while it increased inlet temperature and thermal efficiency. Increasing *φ* improved temperature change, solar irradiation, and thermal efficiency.
Khashan et al. [89]	Fe_3_O_4_-SiO_2_/DIW	1 mg/mL and 2 mg/mL	DASC	Fe_3_O_4_- 7.8 SiO_2_-50	DLS	-	After 5 min of irradiation, the photothermal efficiency of 65.6%, 85.4%, and 98.5% was attained with DIW, kerosene + 2 mg/mL Fe_3_O_4_-SiO_2_ NF, and kerosene + 1 mg/mL Fe_3_O_4_-SiO_2_ (1 mg/mL) NF, respectively.
Yu and Xuan [91]	CuO-Ag (8:2 and 7:3)/DIW	0.15–0.25%	DASC	-	UV	-	At a volume fraction of 0.025% and irradiation of 7000 s, peak temperature change and photothermal efficiency of 34.1 °C and 96.11%, respectively, were reached using CuO-Ag (7:3)/DIW NF as a thermal fluid.
Fang and Xuan [90]	CuO-ZnO (70:30 and 50:50)/DIW	0.001–0.01%	DASC		UV, Visual	κ	At *φ* = 0.01%, the maximum solar absorption efficiency of 99.47% (CuO), 98.67% and (CuO-ZnO (70:30)), and 94.78% (CuO-ZnO (50:50)) were obtained, respectively. Maximum photothermal efficiency of 97.4% (30 °C) and 34.7% (70 °C) was reported for CuO-ZnO (70:30)/DIW NFs.
Farajzadeh et al. [123]	Al_2_O_3_-TiO_2_ (1:1)/DIW	0.1 wt% and 0.2 wt%	FPSC	Al_2_O_3_-20 TiO_2_-15	Visual, CTAB	-	The highest thermal efficiency was recorded using Al_2_O_3_-TiO_2_/DIW NF at a flow rate of 2 L/m and 0.2 wt%. At 0.1 wt%, efficiencies of 19%, 21%, and 26% were obtained for TiO_2_, Al_2_O_3_, and Al_2_O_3_-TiO_2_ NFs respectively, compared with DIW.
Qu et al. [109]	GO-MWCNT/Therminol^®^ 66	10–150 ppm	DASC	GO- 0.5–5 μm MWCNT-20–30	UV, Oleic acid	Extinction coefficient, absorbance, and transmittance.	Under indoor and outdoor conditions, the temperature of 100 ppm-GO-MWCNT/therminol^®^66 NF was 94 °C and 153 °C and 11.6 °C and 97 °C higher than therminol^®^66, respectively. The collector efficiency of 100 ppm HNF was 97% and 70%, respectively.
Sundar et al. [85]	ND-Co_3_O_4_ (67:33)/DW	0.05–0.15 wt%	FPSC	-	Visual	κ and μ	At a flow rate of 1.35 L/min, peak Nu, h, f, and collector efficiency of 21.23%, 36.41%, 1.13-fold, and 59.78% were attained using 0.15 wt% ND-Co_3_O_4_ nano-coolant, respectively. A collector efficiency of 49.81% was obtained for DW.

## Data Availability

Not applicable.

## Nomenclature

ZP—Zeta potential	UV—ultraviolet
wt%—percent weight	PVP—polyvinylpyrrolidone
EG—ethylene glycol	SDBS—sodium dodecyl benzene sulfonate
DLS—dynamic light scattering	SDS—sodium dodecyl sulfate
PG—polyethene glycol	SLS—sodium lauryl sulfate
SHMP—sodium hexa meta phosphate	CTAB—centrimonium bromide
c_p_—specific heat capacity, kJ/kg K	DASC—direct absorption solar collector
DIW—deionized water	DW—distilled water
EC—extinction coefficient	EGR—entropy generation rate, W/K
EO—engine oil	f— friction factor
FLH—freezing latent heat, kJ/kg	FPSC—flat plate solar collector
FT or ST—freezing or solidification temperature, °C	h—heat transfer coefficient, W/m^2^ K
HNF—hybrid nanofluid	HNP—hybrid nanoparticles
MLH—melting latent heat, kJ/kg	MNF—mono nanofluid
MT—meting temperature, °C	NF—nanofluid
NP—nanoparticles	PCM—phase change materials
PEC—performance evaluation criteria	PTEC—photo-thermal energy conversion
PV/T—photovoltaic-thermal	SAR—specific absorption rate, W/μl
SWEA—solar weighted energy	W—water
Greek symbols	
*φ*—volume or weight concentration or fraction	κ—thermal conductivity, W/m K
*ρ*—density, kg/m^3^	θ_c_—contact angle, °
μ—viscosity, mPas	
